# Dysregulation of plasma circulating microRNAs in all-cause and cause-specific cancers: the Rotterdam Study

**DOI:** 10.1186/s40364-024-00626-5

**Published:** 2024-08-13

**Authors:** Yu Shuai, Xiaofang Zhang, Birgit D. A. Lavrijssen, M. Arfan Ikram, Rikje Ruiter, Bruno Stricker, Mohsen Ghanbari

**Affiliations:** 1 Department of Epidemiology, Erasmus MC, University Medical Center, P.O. Box 2040, Rotterdam, 3000 CA The Netherlands; 2 Department of Surgery, Erasmus MC, University Medical Center, Rotterdam, the Netherlands; 3 Department of Internal Medicine, Maasstad Hospital, Rotterdam, the Netherlands

**Keywords:** Circulatory miRNAs, Biomarker, Common cancers, Hematologic tumors, Population-based study

## Abstract

MicroRNAs (miRNAs) are small non-coding RNAs involved in post-transcriptional regulation of gene expression. Mounting evidence underscores the dysregulation of miRNAs to be associated with cancer development and progression by acting as tumour suppressors and oncogenes. However, their potential as biomarkers for early diagnosis of different cancers remains incompletely unraveled. We explored the relationship between plasma circulatory miRNAs and cancer risk within the population-based Rotterdam Study cohort. Plasma samples were collected at baseline (between 2002 and 2005) and miRNA levels were measured in 1,999 participants, including 169 prevalent cancer cases. The occurrence of cancer was assessed by continuous monitoring of medical records in 1,830 cancer-free participants until January 1, 2015. We assessed the association between incidence of five common cancers (blood, lung, breast, prostate, and colorectal) and 591 miRNAs well-expressed in plasma, using adjusted Cox proportional-hazards regression models. Our longitudinal analysis identified 13 miRNAs significantly associated with incident hematologic tumors surpassing the Bonferroni-corrected *P* < 8.46 × 10^− 5^, 12 of them (miR-6124, miR-6778-5p, miR-5196, miR-654-5p, miR-4478, miR-4430, miR-4534, miR-1915-3p, miR-4644, miR-4292, miR-7111-5p, and miR-6870-5p) were also associated with prevalent hematologic tumors in the cross-sectional analysis at the baseline. In-silico analyses of the putative target genes of 13 identified miRNAs highlighted relevant genes and pathways linked to hematologic tumors. While no significant miRNA association was found for other four studied cancers, two miRNAs (miR-3157-5p and miR-3912-5p) showed nominal association with incident of three different cancer types. Overall, this study indicates that plasma levels of several miRNAs are dysregulated in hematologic tumors, highlighting their potential as biomarkers for early diagnosis as well as being involved in the pathogenesis of blood cancers.

**Supplementary Information:**

The online version contains supplementary material available at 10.1186/s40364-024-00626-5.

**To the editor**.

MicroRNAs (miRNAs) and the molecules involved in their biogenesis, including miRNA biogenesis enzymes, can function as oncogenes or tumor suppressor genes, playing regulatory roles in cancer. [1, 2] However, the diagnostic accuracy of miRNAs as potential biomarkers for early cancer detection varies across different cancer types, contributing to the complexity of the field [3, 4]. Hence, we conducted a study to systematically investigate the association between 591 well-expressed extracellular miRNAs in plasma and the incidence of cancer, using data from the prospective, population-based Rotterdam Study (RS) cohort. Our aim was to identify plasma miRNAs with potential as biomarkers for cancer [5]. Post-hoc analyses were also conducted on the putative target genes of identified miRNAs to gain insights into the relevant molecular pathways through which they could contribute to cancer pathogenesis. Participants characteristics, including baseline prevalence of cancer and incidence of cancer during follow-up, are outlined in Table S1. Methods are detailed in Additional file (Supplementary Methods).

Over a mean follow-up period of 8.8 (± 3.1), 311 out of 1,830 individuals were diagnosed with any cancer. Our longitudinal analysis showed that plasma levels of 13 miRNAs were significantly associated with the incidence of hematological tumors (33 cases and 1,519 controls), passing the Bonferroni-corrected threshold of *P* < 8.46 × 10^− 5^ in the fully-adjusted model (Table 1, Fig. 1A). No significant association was found between miRNA levels and other studied cancers.Yet, we found 86, 42, 19, 19, 77 and 19 miRNAs that were nominally (*P* < 0.05) associated with hematological, lung, breast, colorectal, prostate and all-cause cancer, respectively (Figure S1, Table S2-7). Additionally, we checked whether common miRNAs are associated with different cancer types and found miR-3157-5p and miR-3912-5p that were recurrent across three cancer types (blood, breast, and prostate), at the level of nominal association (*P < 0.05*) (Table S8, Figure S2). We also did cross-sectional study and found that 12 of the 13 identified miRNAs were significantly associated with prevalent hematologic tumors at baseline (11 cases and 1,830 controls) in the fully-adjusted model(*P* < 3.85 × 10^− 3^, 0.05/13 miRNAs, Table 1, Table S9). The Wilcoxon test was employed to examine differences in plasma expression patterns of 13 identified miRNAs in patients with incident hematologic tumors group and normal (cancer-free) group. In general, the tumor group exhibited higher expression levels of miR-1915-3p, miR-4430, miR-4478, miR-4505, miR-4644, miR-6124, miR-654-5p, miR-6778-5p, and miR-7111-5p compared to the normal group (Fig. 1B).

**Fig. 1:**
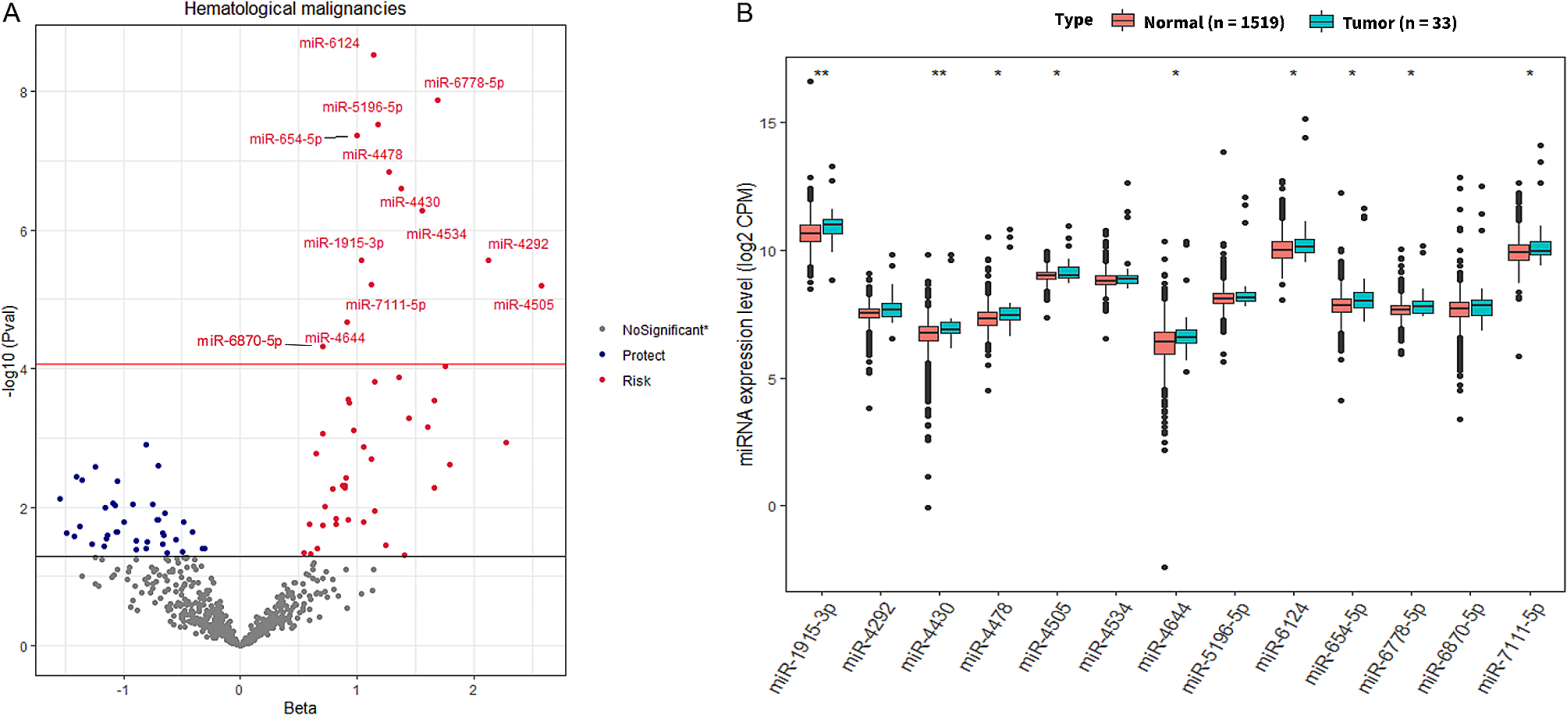
Association of plasma microRNA levels with incident hematological tumors. (**A**) The Volcano plots depict the results from the Cox proportional hazards regression model. Red dots indicate risk miRNAs that are at least nominally associated. Blue dots are protective miRNAs at least nominally associated. Grey dots refer to miRNAs with no significant association at *P* < 0.05. Significantly associated miRNAs are labelled by name at Bonferroni-correction *P* < 8.46 × 10^− 5^. Black lines indicate *P* < 0.05, red lines refer to *P* < 8.46 × 10^− 5^. (**B**) Comparison between plasma levels of the 13 miRNAs in 33 patients with incident hematologic tumors and 1,519 healthy participants (cancer-free). ** means *P* < 0.01; * means *P* < 0.05. Hematologic tumors refers to lymphatic and hematopoietic malignancies. Abbreviations: miRNAs, microRNAs; CPM: counts per million

**Table 1:** Plasma levels of 13 extracellular miRNAs associated with risk of hematologic tumors

miRNA ID	Longitudinal studies (incident)			Cross-sectional studies (prevalent)		
	HR	95% CI	*P* value	Beta	SE	*P* value
miR-6124	3.13	2.15–4.57	2.94 × 10^− 09^	1.12	0.27	**3.15 × 10** ^**− 05**^
miR-6778-5p	5.40	3.02–9.65	1.32 × 10^− 08^	2.00	0.49	**3.76 × 10** ^**− 05**^
miR-5196-5p	3.24	2.14–4.95	2.95 × 10^− 08^	1.14	0.30	**1.48 × 10** ^**− 04**^
miR-654-5p	2.72	1.90–3.89	4.27 × 10^− 08^	1.31	0.31	**2.22 × 10** ^**− 05**^
miR-4478	3.57	2.22–5.74	1.45 × 10^− 07^	1.69	0.40	**2.17 × 10** ^**− 05**^
miR-4430	3.96	2.35–6.68	2.50 × 10^− 07^	1.75	0.41	**1.75 × 10** ^**− 05**^
miR-4534	4.74	2.58–8.70	5.27 × 10^− 07^	1.47	0.42	**4.98 × 10** ^**− 04**^
miR-1915-3p	2.81	1.83–4.33	2.73 × 10^− 06^	1.12	0.35	**1.25 × 10** ^**− 03**^
miR-4292	8.37	3.44–20.35	2.76 × 10^− 06^	2.20	0.64	**5.90 × 10** ^**− 04**^
miR-7111-5p	3.08	1.89–5.01	6.18 × 10^− 06^	1.56	0.31	**5.85 × 10** ^**− 07**^
miR-4505	13.16	4.30-40.27	6.27 × 10^− 06^	0.76	0.98	4.41 × 10^− 01^
miR-4644	2.50	1.64–3.82	2.16 × 10^− 05^	0.95	0.28	**6.89 × 10** ^**− 04**^
miR-6870-5p	2.03	1.44–2.86	4.73 × 10^− 05^	0.91	0.22	**4.75 × 10** ^**− 05**^

Note: The full model is adjusted for age, sex, sub-cohort, smoking status, body mass index, alcohol consumption, education level, present of chronic diseases, and red blood cells count. Cox proportional hazards regression model was used for the longitudinal study and logistic regression was used for the cross-sectional study. The Bonferroni-corrected significance threshold is 8.46 × 10^− 5^ (0.05/591 miRNAs). The *p* values surpassing the Bonferroni-corrected threshold of 3.85 × 10^− 3^ (0.05/13 miRNAs) are bolded in the cross-sectional studies. The table is sorted based on the Bonferroni-corrected *p* value association of miRNAs with cancer in longitudinal studies in the fully adjusted model. Hematologic tumors refer to hematological lymphatic and hematopoietic malignancies. Abbreviations: HR, hazard ratio; CI, confidence interval; SE, the standard error; miRNA, microRNA

We subsequently retrieved the predicted target genes of the 13 miRNAs associated with incident hematological tumors from the miRWalk open-source platform (focusing on target genes that overlapped in at least two of the miRNA-target prediction databases), resulting in 534 putative target genes for all 13 miRNAs (Table S10). Among these, 31 genes overlapped with those reported previously in the Genome-wide association study of blood tumor traits, and 215 genes were identified among the genes reported by a previous Epigenome-wide association study, which may further indicate the importance of identified miRNAs in pathogenesis of hematologic tumors (Table S11). [6, 7] Next, using miRPathDB 2.0 for KEGG analysis, we found that the putative target genes of 13 hematologic tumor-related miRNAs were linked to numerous cancer-related pathways (Figure S3). These include dysregulation of signaling pathways, such as Epstein-Barr virus infection, MARK, and Ras, which have been linked to the development and progression of multiple hematologic tumors, consistent with the results shown by our KEGG analysis. [8, 9] Finally, we investigated whether the identified miRNAs in our study have been associated with hematologic tumors or other types of cancers in previous studies. A summary of the evidence for these associations is presented in Table S12. Despite the lack of consensus on the methods and tissues, we were able to replicate some of the findings from previous studies on hematological tumors. For instance, Di et al. [10]. found that 117 out of 470 studied miRNAs were differentially expressed in mantle cell lymphoma. Of these, the higher expression level of miR-654 precursor was replicated in our study. Additionally, the higher plasma levels of two miRNAs (miR-5196 and miR-4430) significantly associated with incident hematologic tumors in our study were previously linked to acute lymphoblastic leukemia and multiple myeloma (MM), respectively [11, 12]. These observations may suggest that some of the identified miRNAs influence the pathophysiology of the blood tumors through alterations in the expression of highlighted genes that warrant further molecular investigations.

Collectively, this study indicates significant associations between plasma levels of several miRNAs and the risk of hematological tumors. These miRNAs may hold promise as potential biomarkers for early diagnosis of hematologic tumors and might also be involved in the pathogenesis of these cancers that warrant further studies. Subsequent investigations should incorporate larger sample sizes and in vitro experimental validation studies to replicate and confirm the potential of the identified miRNAs as biomarkers and/or drivers of hematologic tumors.

### Electronic supplementary material

Below is the link to the electronic supplementary material.

**Supplementary Material 1:Supplementary Tables.Table S1** provides Baseline characteristics of the Rotterdam Study participants of this study. **Table S2** the nominal association results of Cox proportional hazard models analyzing miRNAs and incident hematological tumors. **Table S3** provides the nominal association results of Cox proportional hazard models analyzing miRNAs and incident lung cancer. **Table S4** provides the nominal association results of Cox proportional hazard models analyzing miRNAs and incident breast cancer. **Table S5** provides the nominal association results of Cox proportional hazard models analyzing miRNAs and incident colorectal cancer. **Table S6** provides the nominal association results of Cox proportional hazard models analyzing miRNAs and incident prostate cancer. **Table S7** provides the nominal association results of Cox proportional hazard models analyzing miRNAs and incident all causes cancer. **Table S8** presents the list of nominally associated with incidence of three different cancer types. **Table S9** provides the results for the logistic regression analysis between miRNAs and incident hematological tumors. **Table S10** provides the list of target genes for the 13 hematologic tumor associated miRNAs. **Table S11** shows the miRNA target genes that were previously identified in association with hematological tumors in GWAS and EWAS. **Table S12** shows supporting evidence for the association between the 13 hematological tumor-related miRNAs and various cancer types as reported in previous studies. **Supplementary Figures**. **Figure S1** provides a volcano plot depicting the association between microRNA levels and different incident cancers; **Figure S2** presents a venn diagram of miRNAs nominally associated (*P* < 0.05) with various incident cancers; **Figure S3** depicts the enrichment plot for the 13 identified hematological tumor-associated miRNAs in the KEGG pathways.

## Data Availability

Rotterdam Study data can be made available to interested researchers upon request. Requests can be directed to data manager Frank J.A. van Rooij (f.vanrooij@erasmusmc.nl). We are unable to place data in a public repository due to legal and ethical restraints. Sharing of individual participant data was not included in the informed consent of the study, and there is potential risk of revealing participants’ identities as it is not possible to completely anonymize the data.

## Abbreviations

MiRNAs: MicroRNAs
RS: Rotterdam Study
