# Supplementary material for: Dysregulation of plasma circulating microRNAs in all-cause and cause-specific cancers: the Rotterdam Study
Source: Biomark Res. 2024 Aug 13;12:83. doi: 10.1186/s40364-024-00626-5 (PMC11321125; doi:10.1186/s40364-024-00626-5)
Supplement: Supplementary file 1 — Supplementary Material 1:Supplementary Tables.Table S1 provides Baseline characteristics of the Rotterdam Study participants of this study. Table S2 the nominal association results of Cox proportional hazard models analyzing miRNAs and incident hematological tumors. Table S3 provides the nominal association results of Cox proportional hazard models analyzing miRNAs and incident lung cancer. Table S4 provides the nominal association results of Cox proportional hazard models analyzing miRNAs and incident breast cancer. Table S5 provides the nominal association results of Cox proportional hazard models analyzing miRNAs and incident colorectal cancer. Table S6 provides the nominal association results of Cox proportional hazard models analyzing miRNAs and incident prostate cancer. Table S7 provides the nominal association results of Cox proportional hazard models analyzing miRNAs and incident all causes cancer. Table S8 presents the list of nominally associated with incidence of three different cancer types. Table S9 provides the results for the logistic regression analysis between miRNAs and incident hematological tumors. Table S10 provides the list of target genes for the 13 hematologic tumor associated miRNAs. Table S11 shows the miRNA target genes that were previously identified in association with hematological tumors in GWAS and EWAS. Table S12 shows supporting evidence for the association between the 13 hematological tumor-related miRNAs and various cancer types as reported in previous studies. Supplementary Figures. Figure S1 provides a volcano plot depicting the association between microRNA levels and different incident cancers; Figure S2 presents a venn diagram of miRNAs nominally associated (P < 0.05) with various incident cancers; Figure S3 depicts the enrichment plot for the 13 identified hematological tumor-associated miRNAs in the KEGG pathways. [file 40364_2024_626_MOESM1_ESM.docx]

# **Dysregulation of plasma circulating microRNAs in all-cause and cause-specific cancers: the Rotterdam Study**

Yu Shuai, Xiaofang Zhang, Birgit D A Lavrijssen, M. Arfan Ikram, Rikje Ruiter, Bruno Stricker, Mohsen Ghanbari

**SUPPLEMENTARY MATERIALS AND METHODS**

**INDEX**

**Supplementary Methods……..………………………………….…....….….…………….…....…...…...….page 2**

**Supplementary Tables.…………………….…....…...…..………...….….…….…….………..…....…….....page 6**

**Supplementary Figures.………………………….…....…...….….…....…..……………….…....…...….….page 40**

**Supplementary References.………………………….…....…...….….……………….….. ...…...…...….….page 46**

**Supplementary Methods**

**Study population**

The study was carried out in the Rotterdam Study (RS), which is a prospective population-based cohort consisting of individuals aged 45 years or older, residing in the suburb of Ommoord, Rotterdam, the Netherlands. In 1990, the initial cohort of study participants (RS-I) comprised 7,983 residents aged 55 years or older. In 2000, the second cohort (RS-II) was expanded to include an additional 3 011 participants who had moved to Ommoord or turned 55 years old. A detailed description of the Rotterdam Study can be found elsewhere [1]. The current study examined plasma expression profiles of circulating miRNAs in two random subsets of these cohorts: a group of 1 000 participants from the fourth visit to RS-I (RS-I-4), and a group of 1 000 participants from the second visit to RS-II (RS-II-2). These visits in the Rotterdam Study were conducted between 2002 and 2005 and received regular follow-ups at intervals of approximately 3 to 5 years. Among the 2,000 participants with miRNA data, one participant was excluded due to missing profiling data on all miRNAs. Furthermore, 169 participants with prevalent cancer, who had any type of cancer at the time of the baseline assessment (2002-2005), were excluded, resulting in a total sample size of 1,830 (cancer-free) participants for the longitudinal analysis.

**MiRNA expression profiling**

Blood samples were collected in tubes treated with ethylenediaminetetraacetic acid (EDTA) and then centrifuged. The resulting plasma was aliquoted and frozen at -80 °C following standard procedures. Plasma levels of cell-free miRNAs were determined using the HTG EdgeSeq miRNA Whole Transcriptome Assay (WTA), which measures the expression of 2083 human mature miRNAs. The WTA characterizes miRNA expression patterns, and measures the expression of 13 housekeeping genes, that allows flexibility in data normalization and analysis. Plasma samples, for two re-measurements that generally is sufficient to obtain a valid result for all samples, were sent to HTG Molecular Diagnostics (AZ, USA) for sequencing. Each sample was tagged individually with molecular barcodes, tagged samples were pooled and sequenced on an Illumina NextSeq 500 sequencer (Illumina, San Diego, CA, USA). Quantification of miRNA expression was based on counts per million (CPM). The log2 transformation of CPM was used as standardization and adjustment for total reads within each sample. The miRNAs with log2 CPM< 1.0 were considered as not expressed in the samples. Moreover, we focused on 591 miRNAs that were expressed at good levels in plasma. These 591 (well-expressed) miRNAs are those with >50% values above Lower Limit of Quantification (LLOQ). The LLOQ was determined through a statistical approach involving a monotonic decreasing spline curve fitted to the log2-transformed CPM values of all miRNAs, using their means and standard deviations. This curve helped establish a threshold where miRNAs with sufficient expression consistency above the LLOQ were considered reliably quantifiable.[2]

**Assessment of cancer**

Cancer case data were obtained from the general practitioners' records and cross-referenced with the Rotterdam PATHAN pathology laboratory, the national PALGA pathology database, and a hospital discharges database. Cancer, in this context, was defined as any primary malignant tumor, excluding non-melanoma skin cancer cases. The analysis specifically included only pathology-confirmed cancer cases to minimize the risk of false-positive diagnoses. Two independent physicians coded and classified cancer diagnoses according to the International Classification of Diseases, 10th revision (ICD-10).[3] These include lung cancer (C34), breast cancer (C50), colorectal cancer (C18-C20), prostate cancer (C61), and hematological tumors (C81-C96). In cases of discrepancies, consensus was achieved through consultation with a physician specialized in internal medicine. The date of diagnosis was determined based on the biopsy date for solid tumors, laboratory assessment for hematologic tumors, or, if unavailable, the date of hospital admission or discharge letter. In case of multiple cancers within one participant, only the first diagnosis was considered for analysis. The follow-up for incident cancer extended until January 1, 2015. Participants were tracked from the commencement of the study until the occurrence of cancer, death, the last health status update indicating being cancer-free, or January 1, 2015, whichever transpired first

**Assessment on covariates**

Home administered interviews were used to assess participants’ age, sex and smoking status (current/former/never). Weight and height were measured when participants were standing without heavy outer garments or shoes. Body mass index (BMI) was calculated based on weight in kilograms divided by the height in meters squared (kg/m^2^). Participants were categorized as primary education (primary), lower/intermediate general education or lower vocational education (lower), intermediate vocational education or higher general education (intermediate), and higher vocational education or university (higher). Alcohol consumption was assessed in grams of ethanol per day. Participants with hypertension, diabetes mellitus (DM), coronary heart disease (CHD) (myocardial infarction, percutaneous coronary intervention, or coronary artery bypass grafting), stroke, dementia and chronic obstructive pulmonary disease (COPD) were considered to have chronic diseases (yes/no). History of chronic diseases were assessed by interview and verified by reviewing medical records. In addition, the same participant may have more than two chronic diseases. Blood pressure was measured twice in a seated position on the right arm using a random-zero sphygmomanometer, and the average of the two measurements was recorded. Hypertension was defined as a systolic blood pressure (BP) ≥ 140 mm Hg or a diastolic BP ≥ 90 mm Hg, or the use of antihypertensive medication prescribed for hypertension.[4] Prevalent type 2 DM was identified based on the World Health Organization criteria, including fasting glucose levels ≥7.0 mmol/L, nonfasting glucose levels ≥11.1 mmol/L, or the use of glucose-lowering medication.[5] CHD was defined as a participant having a history of myocardial infarction or undergoing coronary artery bypass grafting or percutaneous coronary revascularization procedure.[6] Stroke was defined as the occurrence of a rapidly developing syndrome characterized by focal or global disturbance of cerebral function, with symptoms lasting 24 hours or longer or leading to death, and with no apparent cause other than vascular origin, in accordance with the World Health Organization definition.[7] Participants were screened for dementia at baseline and subsequent center visits with the Mini-Mental State Examination and the Geriatric Mental Schedule organic level. Those with a Mini-Mental State Examination score < 26 or Geriatric Mental Schedule score > 0 underwent further investigation and informant interview, including the Cambridge Examination for Mental Disorders of the Elderly. Blood samples of participants were obtained during the visit to the research center. Red blood cell (RBC) counts (10^12^/L) and white blood cell (WBC) counts (10^9^/L) measured using the COULTER® Ac·T diff2™ Hematology Analyzer (Beckman Coulter, San Diego, California, USA).

Statistical analyses

For the longitudinal study, we implemented Cox proportional hazard models using calendar time as the time scale to determine hazard ratios (HR) with 95% confidence intervals (CIs) for the association between plasma miRNA levels (log2 CPM) and cancer risk, including all-cause cancer and cause-specific cancer (five common cancers). The Bonferroni-corrected *P*-value threshold was calculated based on the number of tested miRNAs (0.05/591 = 8.46 × 10^−5^). The basic model (Model 1) was adjusted for age, sex, and cohort, whereas the multivariable model (Model 2) was further adjusted for BMI, smoking status, alcohol consumption, education level, chronic diseases, RBC and WBC counts. No WBC adjustment was made for hematological tumors. Importantly, sex was not adjusted for in models 1 and 2 for breast and prostate cancers, as these analyses were restricted to female and male participants, respectively.

For the cross-sectional study, multivariable logistic regression models were employed to explore the association between plasma levels of miRNAs identified by the longitudinal analysis and the prevalent of related cancer at baseline. Since there was a smaller number of cancer cases at baseline and due to lower statistical power, this analysis was conducted only for miRNAs identified by the longitudinal analysis.

To mitigate potential bias resulting from missing values, missing covariate data (ranging from 0.2% to 4.2%) were imputed using a multiple imputation technique (n = 5 imputations). All analyses were conducted using SPSS statistical software (version 25; IBM Corp) and R software (version 4.0.5; The R Foundation for Statistical Computing). Volcano plots, heatmaps, and Venn diagrams were generated to visualize the associations between plasma miRNA levels and various cancers. Furthermore, the Wilcoxon rank-sum test was used to compare the expression of identified miRNAs between the normal and tumor groups. The R packages ggplot2, pheatmap, and ggrepel were employed for these analyses.

To investigate the potential involvement of cancer-associated miRNAs in underlying disease pathways, we conducted an in-silico analysis of their putative target genes. To retrieve predicted target genes of miRNAs, we utilized the open-source platform miRWalk,[8] which incorporates three commonly used miRNA target prediction databases (TargetScan,[9] miRDB,[10] and miRTarBase[11]). We focused on the target genes overlapping in at least 2 out of the three databases. Next, we conducted a literature search and utilized various web tools such as EWAS Atlas [12] and GWAS catalogs (using trait ID, MONDO_0002334) [13], to explore whether any of the relevant miRNAs and their putative target genes have been reported previously to be associated with cancer. Lastly, Gene Set Enrichment Analysis (GSEA) in miRPathDB 2.0 [14] were used to explore the Kyoto Encyclopedia of Genes and Genomes (KEGG) pathway of miRNA target genes, respectively.

**Supplementary Tables**

| **Table** | **Title** |
| --- | --- |
| Table S1 | Baseline characteristics of the Rotterdam Study participants of this study |
| Table S2 | The list of miRNAs nominally associated with incident hematological tumors |
| Table S3 | The list of miRNAs nominally associated with incident lung cancer |
| Table S4 | The list of miRNAs nominally associated with incident breast cancer |
| Table S5 | The list of miRNAs nominally associated with incident colorectal cancer |
| Table S6 | The list of miRNAs nominally associated with incident prostate cancer |
| Table S7 | The list of miRNAs nominally associated with incident all causes cancer |
| Table S8 | Two miRNAs nominally associated with incidence of three different cancer types |
| Table S9 | The list of miRNAs nominally associated with prevalent hematological tumors |
| Table S10 | Putative target genes of the thirteen hematologic tumors associated miRNAs |
| Table S11 | Putative target genes of the 13 hematological tumor-related miRNAs previously linked with hematological tumors through (epi)genomic studies |
| Table S12 | Supporting evidence for the association between the 13 hematological tumor-related miRNAs and different cancer types reported in previous studies |

**Table S1. Baseline characteristics of the Rotterdam Study participants of this study**

| **Characteristic** | **Prevalent cancer** (n =169) | **Incident cancer** (n=311) | **No cancer** (n=1519) |
| --- | --- | --- | --- |
| **Age, mean (±SD), y** | 73.1 (6.9) | 71.2 (6.8) | 71.5 (7.77) |
| **Sex, Male, n (%)** | 85 (50.3) | 176 (56.6) | 597 (39.3) |
| **Rotterdam Study cohort, RS-I, n (%)** | 96 (56.8) | 168 (54.0) | 736 (48.5) |
| **Smoking status, n (%)** |  |  |  |
| Current | 23 (13.9) | 56 (18.3) | 185 (12.3) |
| Former | 97 (58.8) | 173 (56.5) | 818 (54.5) |
| Never | 45 (27.3) | 77 (25.2) | 497 (33.1) |
| **BMI, kg/m^2^, mean (±SD)** | 26.7 (3.3) | 27.7 (3.9) | 27.6 (4.2) |
| **Education level, n (%)** |  |  |  |
| Primary | 20 (12.1) | 22 (7.2) | 172 (11.5) |
| Lower | 75 (45.5) | 135 (44.0) | 672 (44.9) |
| Intermediate | 47 (28.5) | 99 (32.2) | 433 (28.9) |
| Higher | 23 (13.9) | 51 (16.6) | 219 (14.6) |
| **Prevalent chronic diseases, n (%)** |  |  |  |
| Hypertension, Yes | 132 (78.1) | 245 (79) | 1173 (79.3) |
| DM, Yes | 24 (15) | 45 (15) | 156 (10.5) |
| CHD, Yes | 17 (10.1) | 37 (12) | 210 (10.7) |
| Stroke, Yes | 5 (3) | 17 (5.5) | 60 (4) |
| Dementia, Yes | 1 (0.6) | 0 (0) | 12 (0.7) |
| COPD, Yes | 12 (7.1) | 25 (8) | 118 (7.8) |
| All, Yes | 137 (81.5) | 256 (82.6) | 1232 (81.8) |
| **Blood Indicators, mean (±SD)** |  |  |  |
| RBC, 10^12/L | 4.7 (0.5) | 4.8 (0.5) | 4.7 (0.4) |
| WBC, 10^12/L | 7.1 (4.7) | 7.3 (5.8) | 6.6 (1.9) |
| **Age at time of cancer diagnosis,**  **mean (±SD), y** | 66.2 (9.2) | 76.5(7.3) | - |
| **Alcohol intake (g/day),** **Median (IQR)** | 7.3 (18.9) | 10 (20.2) | 6.4 (17.9) |
| **Cancer types, yes, n (%)** |  |  |  |
| Colorectal cancer | 24 (14.2) | 43 (13.8) | - |
| Lung cancer | 4 (2.4) | 37 (11.8) | - |
| Breast cancer | 39 (23.1) | 40 (12.8) | - |
| Prostate cancer | 50 (29.6) | 55 (17.7) | - |
| Hematologic tumors | 11 (6.5) | 33 (10.6) | - |
| Other cancer | 41 (24.3) | 103 (33.1) | - |
| Total with cancer | 169 | 311 | - |

Note: Values are represented as mean (±standard deviation), sample sizes (%), or median (inter-quartile range) for characteristics with skewed distributions. Number of missing values for final study population: 28 (1.4%) for smoking status, 31 (1.6%) for education level, 28 (1.4%) for alcohol, 3 (0.2%) for BMI, 9 (0.5%) for chronic disease, 84 (4.2%) for DM, 6 (0.3%) stroke, 14 (0.7%) for dementia, 84 (4.2%) for CHD, 40 (2%) for COPD, 4 (0.2%) for WBC, and 3 (0.2%) for RBC. Hematologic tumors refers to hematological (lymphatic and hematopoietic) tumors. Other cancer means the sum of different cancer samples with a sample size less than 30.

Abbreviations: BMI, body mass index; DM, diabetes mellitus; CHD, coronary heart disease; COPD, chronic obstructive pulmonary disease; RBC, red blood cells; WBC, white blood cells; SD, standard deviation; IQR, interquartile range; y, years; n, number.

**Table S2. The list of miRNAs nominally associated with incident hematological tumors**

| miRNA ID | Model 1 | | | Model 2 | | |
| --- | --- | --- | --- | --- | --- | --- |
|  | HR | 95% CI | *P* value | HR | 95% CI | *P* value |
| miR-6124 | 2.84 | 2.00-4.03 | 4.68×10^-09^ | 3.13 | 2.15-4.57 | 2.94×10^-09^ |
| miR-6778-5p | 5.07 | 2.94-8.72 | 4.89×10^-09^ | 5.40 | 3.02-9.65 | 1.32×10^-08^ |
| miR-4478 | 3.35 | 2.15-5.23 | 9.64×10^-08^ | 3.57 | 2.22-5.74 | 1.45×10^-07^ |
| miR-654-5p | 2.44 | 1.75-3.40 | 1.27×10^-07^ | 2.72 | 1.90-3.89 | 4.27×10^-08^ |
| miR-4430 | 3.67 | 2.26-5.95 | 1.31×10^-07^ | 3.96 | 2.35-6.68 | 2.50×10^-07^ |
| miR-5196-5p | 2.78 | 1.90-4.07 | 1.57×10^-07^ | 3.24 | 2.14-5.92 | 2.95×10^-08^ |
| miR-4534 | 4.36 | 2.44-7.79 | 6.99×10^-07^ | 4.74 | 2.58-8.70 | 5.27×10^-07^ |
| miR-4292 | 6.47 | 2.96-14.15 | 2.86×10^-06^ | 8.37 | 3.44-20.35 | 2.76×10^-06^ |
| miR-4505 | 13.15 | 4.39-39.41 | 4.22×10^-06^ | 13.16 | 4.30-40.27 | 6.27×10^-06^ |
| miR-1915-3p | 2.47 | 1.66-3.68 | 8.19×10^-06^ | 2.81 | 1.83-4.33 | 2.73×10^-06^ |
| miR-7111-5p | 2.85 | 1.79-4.55 | 1.12×10^-05^ | 3.08 | 1.89-5.01 | 6.18×10^-06^ |
| miR-4644 | 2.36 | 1.59-3.49 | 1.83×10^-05^ | 2.50 | 1.64-3.82 | 2.16×10^-05^ |
| miR-765 | 3.61 | 1.88-6.93 | 1.11×10^-04^ | 3.87 | 1.93-7.76 | 1.32×10^-04^ |
| miR-6870-5p | 1.86 | 1.35-2.55 | 1.20×10^-04^ | 2.03 | 1.44-2.86 | 4.73×10^-05^ |
| miR-4800-5p | 5.33 | 2.23-12.70 | 1.61×10^-04^ | 5.28 | 2.15-12.98 | 2.86×10^-04^ |
| miR-155-5p | 5.15 | 2.16-12.28 | 2.15×10^-04^ | 5.78 | 2.40-13.92 | 9.34×10^-05^ |
| miR-1290 | 2.46 | 1.51-4.00 | 2.84×10^-04^ | 2.54 | 1.53-4.22 | 3.15×10^-04^ |
| miR-3940-5p | 2.30 | 1.46-3.61 | 3.03×10^-04^ | 2.53 | 1.53-4.17 | 2.76×10^-04^ |
| miR-6845-5p | 2.82 | 1.59-5.01 | 3.82×10^-04^ | 3.15 | 1.74-5.71 | 1.52×10^-04^ |
| miR-3648 | 1.99 | 1.34-2.95 | 6.36×10^-04^ | 2.03 | 1.34-3.09 | 8.58×10^-04^ |
| miR-4707-3p | 4.01 | 1.78-9.02 | 7.78×10^-04^ | 4.23 | 1.87-9.55 | 5.22×10^-04^ |
| miR-4459 | 2.87 | 1.54-5.37 | 9.54×10^-04^ | 2.88 | 1.51-5.49 | 1.34×10^-03^ |
| miR-4723-3p | 8.80 | 2.41-32.15 | 1.01×10^-03^ | 9.74 | 2.47-38.36 | 1.14×10^-03^ |
| miR-4640-3p | 4.14 | 1.76-9.73 | 1.11×10^-03^ | 4.96 | 1.97-12.52 | 6.91×10^-04^ |
| miR-7851-3p | 2.53 | 1.44-4.44 | 1.19×10^-03^ | 2.63 | 1.50-4.62 | 7.66×10^-04^ |
| miR-381-3p | 0.50 | 0.33-0.77 | 1.65×10^-03^ | 0.50 | 0.32-0.78 | 2.50×10^-03^ |
| miR-126-3p | 0.33 | 0.16-0.67 | 2.11×10^-03^ | 0.35 | 0.17-0.72 | 4.22×10^-03^ |
| miR-6870-3p | 0.50 | 0.32-0.78 | 2.22×10^-03^ | 0.45 | 0.27-0.73 | 1.23×10^-03^ |
| miR-920 | 6.31 | 1.90-20.92 | 2.61×10^-03^ | 6.02 | 1.89-19.19 | 2.41×10^-03^ |
| miR-4270 | 2.86 | 1.44-5.66 | 2.68×10^-03^ | 3.06 | 1.51-6.23 | 2.00×10^-03^ |
| miR-4449 | 1.82 | 1.23-2.71 | 2.83×10^-03^ | 1.92 | 1.28-2.89 | 1.68×10^-03^ |
| miR-4486 | 5.63 | 1.76-18.05 | 2.65×10^-03^ | 5.28 | 1.64-16.99 | 5.29×10^-03^ |
| miR-1275 | 2.42 | 1.33-4.40 | 3.72×10^-03^ | 2.41 | 1.31-4.43 | 4.90×10^-03^ |
| miR-6760-3p | 0.31 | 0.14-0.69 | 3.96×10^-03^ | 0.29 | 0.13-0.65 | 4.64×10^-03^ |
| miR-7150 | 2.32 | 1.29-4.17 | 4.92×10^-03^ | 2.47 | 1.34-4.54 | 3.72×10^-03^ |
| miR-6085 | 2.13 | 1.26-3.61 | 4.93×10^-03^ | 2.20 | 1.26-3.84 | 5.37×10^-03^ |
| miR-4463 | 2.45 | 1.31-4.57 | 5.11×10^-03^ | 2.46 | 1.31-4.59 | 4.93×10^-03^ |
| miR-6873-3p | 0.51 | 0.32-0.82 | 5.25×10^-03^ | 0.53 | 0.32-0.87 | 1.23×10^-02^ |
| miR-648 | 0.25 | 0.10-0.67 | 5.68×10^-03^ | 0.25 | 0.10-0.63 | 3.65×10^-03^ |
| miR-6894-5p | 2.36 | 1.24-4.48 | 8.93×10^-03^ | 2.46 | 1.31-4.62 | 5.29×10^-03^ |
| miR-4279 | 0.28 | 0.10-0.74 | 9.97×10^-03^ | 0.26 | 0.10-0.65 | 4.11×10^-03^ |
| miR-4433b-5p | 0.35 | 0.16-0.78 | 1.01×10^-02^ | 0.34 | 0.15-0.76 | 8.68×10^-03^ |
| miR-6799-5p | 2.05 | 1.18-3.54 | 1.04×10^-02^ | 2.06 | 1.19-3.58 | 9.70×10^-03^ |
| miR-33b-5p | 0.23 | 0.07-0.71 | 1.06×10^-02^ | 0.21 | 0.07-0.66 | 7.57×10^-03^ |
| miR-6796-3p | 0.40 | 0.20-0.81 | 1.14×10^-02^ | 0.40 | 0.15-0.77 | 9.18×10^-03^ |
| miR-1237-5p | 2.41 | 1.21-4.83 | 1.29×10^-02^ | 2.52 | 1.20-5.31 | 1.50×10^-02^ |
| miR-1307-3p | 0.49 | 0.28-0.87 | 1.54×10^-02^ | 0.47 | 0.27-0.83 | 8.99×10^-03^ |
| miR-6769a-3p | 0.70 | 0.52-0.93 | 1.58×10^-02^ | 0.73 | 0.55-0.99 | 3.98×10^-03^ |
| miR-4291 | 0.34 | 0.14-0.82 | 1.64×10^-02^ | 0.31 | 0.13-0.76 | 9.70×10^-03^ |
| miR-5694 | 0.40 | 0.19-0.85 | 1.75×10^-02^ | 0.34 | 0.15-0.77 | 9.49×10^-03^ |
| miR-134-5p | 0.66 | 0.47-0.93 | 1.76×10^-02^ | 0.67 | 0.47-0.94 | 2.28×10^-02^ |
| miR-4728-5p | 2.15 | 1.14-4.04 | 1.81×10^-02^ | 2.28 | 1.17-4.42 | 1.48×10^-02^ |
| miR-3169 | 0.62 | 0.41-0.92 | 1.83×10^-02^ | 0.61 | 0.41-0.91 | 1.64×10^-02^ |
| miR-6775-5p | 1.76 | 1.10-2.83 | 1.89×10^-02^ | 1.81 | 1.11-2.95 | 1.78×10^-02^ |
| miR-30d-5p | 0.23 | 0.07-0.79 | 1.97×10^-02^ | 0.24 | 0.07-0.85 | 2.62×10^-02^ |
| miR-6086 | 1.93 | 1.09-3.40 | 2.38×10^-02^ | 2.03 | 1.13-3.63 | 1.79×10^-02^ |
| miR-3616-5p | 0.52 | 0.29-0.92 | 2.44×10^-02^ | 0.50 | 0.28-0.87 | 1.50×10^-02^ |
| miR-1247-3p | 0.54 | 0.31-0.92 | 2.48×10^-02^ | 0.49 | 0.27-0.87 | 1.53×10^-02^ |
| miR-16-5p | 0.44 | 0.22-0.91 | 2.63×10^-02^ | 0.45 | 0.21-0.96 | 3.90×10^-02^ |
| miR-187-5p | 0.26 | 0.08-0.86 | 2.73×10^-02^ | 0.28 | 0.09-0.91 | 3.37×10^-02^ |
| miR-378e | 2.76 | 1.12-6.81 | 2.78×10^-02^ | 3.16 | 1.30-7.67 | 1.11×10^-02^ |
| miR-326 | 0.51 | 0.28-0.93 | 2.90×10^-02^ | 0.52 | 0.28-0.95 | 3.42×10^-02^ |
| miR-6511a-5p | 0.28 | 0.09-0.88 | 2.90×10^-02^ | 0.25 | 0.08-0.80 | 1.90×10^-02^ |
| miR-6748-3p | 0.58 | 0.36-0.95 | 2.95×10^-02^ | 0.57 | 0.35-0.94 | 2.88×10^-02^ |
| miR-27b-3p | 0.39 | 0.17-0.92 | 3.07×10^-02^ | 0.41 | 0.17-0.96 | 4.08×10^-02^ |
| miR-937-3p | 0.40 | 0.17-0.92 | 3.09×10^-02^ | 0.37 | 0.16-0.83 | 1.65×10^-02^ |
| miR-504-3p | 2.06 | 1.07-3.99 | 3.11×10^-02^ | 2.26 | 1.16-4.44 | 1.72×10^-02^ |
| miR-23a-3p | 0.42 | 0.19-0.93 | 3.15×10^-02^ | 0.41 | 0.18-0.92 | 2.99×10^-02^ |
| miR-374b-3p | 0.32 | 0.12-0.91 | 3.23×10^-02^ | 0.32 | 0.12-0.87 | 2.57×10^-02^ |
| miR-874-3p | 0.39 | 0.16-0.92 | 3.26×10^-02^ | 0.35 | 0.14-0.86 | 2.30×10^-02^ |
| miR-29a-3p | 3.48 | 1.10-10.97 | 3.33×10^-02^ | 3.46 | 1.09-10.97 | 3.46×10^-02^ |
| miR-3157-5p | 0.36 | 0.14-0.92 | 3.36×10^-02^ | 0.35 | 0.14-0.86 | 2.29×10^-02^ |
| miR-4655-3p | 0.25 | 0.07-0.90 | 3.38×10^-02^ | 0.23 | 0.06-0.82 | 2.34×10^-02^ |
| miR-1307-5p | 0.45 | 0.22-0.94 | 3.44×10^-02^ | 0.45 | 0.22-0.93 | 3.14×10^-02^ |
| miR-378d | 2.55 | 1.07-6.07 | 3.47×10^-02^ | 2.87 | 1.21-6.78 | 1.63×10^-02^ |
| miR-6797-5p | 1.92 | 1.04-3.54 | 3.58×10^-02^ | 1.93 | 1.03-3.59 | 3.91×10^-02^ |
| miR-3184-3p | 0.55 | 0.31-0.96 | 3.67×10^-02^ | 0.52 | 0.29-0.92 | 2.55×10^-02^ |
| miR-6742-5p | 0.32 | 0.11-0.96 | 4.11×10^-02^ | 0.31 | 0.10-0.93 | 3.61×10^-02^ |
| miR-339-3p | 0.72 | 0.53-0.99 | 4.14×10^-02^ | 0.72 | 0.53-0.98 | 3.97×10^-02^ |
| miR-3180 | 0.55 | 0.31-0.98 | 4.24×10^-02^ | 0.51 | 0.29-0.91 | 2.34×10^-02^ |
| miR-3912-5p | 0.35 | 0.12-0.99 | 4.83×10^-02^ | 0.32 | 0.11-0.88 | 2.82×10^-02^ |
| miR-4684-3p | 0.63 | 0.40-1.01 | 5.59×10^-02^ | 0.61 | 0.37-0.99 | 4.34×10^-02^ |
| miR-3180-3p | 0.58 | 0.32-1.06 | 7.85×10^-02^ | 0.53 | 0.29-0.99 | 4.53×10^-02^ |
| miR-2861 | 1.60 | 0.96-2.67 | 6.89×10^-02^ | 1.73 | 1.01-2.96 | 4.59×10^-02^ |
| miR-6780b-5p | 1.74 | 0.98-3.09 | 5.67×10^-02^ | 1.82 | 1.01-3.30 | 4.75×10^-02^ |
| miR-29c-3p | 3.47 | 0.90-13.4 | 7.14×10^-02^ | 4.08 | 1.01-16.57 | 4.92×10^-02^ |

Note: Model 1 : adjusted for age, sex, cohort. Model 2: adjusted for model1, smoking status, chronic disease, BMI, RBC, alcohol and education. The table is ranked according to the P values of miRNAs nominally. The table is ranked according to the P values of miRNAs nominally associated (P<0.05) with incident associated (P<0.05) with incident haematological tumors in model 1. Hematologic tumors refers to lymphatic and hematopoietic tumors. Abbreviations. BMI, body mass index; HR, hazard ratio; RBC, red blood cells; miRNA, microRNA; CI, confidence interval.

**Table S3. The list of miRNAs nominally associated with incident lung cancer**

| miRNA ID | Model 1 | | | Model 2 | | |
| --- | --- | --- | --- | --- | --- | --- |
|  | HR | 95% CI | *P* value | HR | 95% CI | *P* value |
| miR-125a-5p | 6.04 | 1.98-18.42 | 1.57×10^-03^ | 5.78 | 1.89-17.65 | 2.07×10^-03^ |
| miR-4795-5p | 0.30 | 0.14-0.67 | 3.19×10^-03^ | 0.34 | 0.15-0.78 | 1.03×10^-02^ |
| miR-99b-5p | 4.84 | 1.67-14.03 | 3.63×10^-03^ | 4.29 | 1.50-12.27 | 6.63×10^-03^ |
| miR-144-5p | 0.49 | 0.30-0.79 | 3.67×10^-03^ | 0.51 | 0.30-0.86 | 1.20×10^-02^ |
| miR-7845-5p | 0.50 | 0.31-0.80 | 4.11×10^-03^ | 0.48 | 0.30-0.78 | 2.84×10^-03^ |
| miR-4753-5p | 0.32 | 0.14-0.72 | 5.78×10^-03^ | 0.37 | 0.16-0.86 | 2.05×10^-02^ |
| miR-1231 | 0.56 | 0.37-0.86 | 7.36×10^-03^ | 0.53 | 0.34-0.81 | 3.45×10^-03^ |
| miR-146b-5p | 8.08 | 1.71-38.20 | 8.41×10^-03^ | 6.10 | 1.36-27.38 | 1.83×10^-02^ |
| miR-6787-5p | 2.43 | 1.21-4.87 | 1.24×10^-02^ | 2.37 | 1.18-4.75 | 1.51×10^-02^ |
| miR-144-3p | 0.55 | 0.35-0.88 | 1.29×10^-02^ | 0.59 | 0.37-0.96 | 3.44×10^-02^ |
| miR-181a-5p | 3.96 | 1.33-11.82 | 1.36×10^-02^ | 3.54 | 1.21-10.40 | 2.12×10^-02^ |
| miR-2861 | 0.48 | 0.27-0.86 | 1.40×10^-02^ | 0.46 | 0.26-0.82 | 8.50×10^-03^ |
| miR-331-3p | 3.04 | 1.25-7.43 | 1.45×10^-02^ | 2.90 | 1.18-7.12 | 2.01×10^-02^ |
| miR-769-5p | 2.13 | 1.15-3.96 | 1.62×10^-02^ | 2.27 | 1.20-4.28 | 1.12×10^-02^ |
| miR-6165 | 0.54 | 0.32-0.90 | 1.81×10^-02^ | 0.55 | 0.32-0.94 | 2.80×10^-02^ |
| miR-149-3p | 1.64 | 1.09-2.47 | 1.87×10^-02^ | 1.58 | 1.01-2.48 | 4.61×10^-02^ |
| miR-23a-5p | 1.88 | 1.10-3.22 | 2.12×10^-02^ | 1.88 | 1.08-3.27 | 2.56×10^-02^ |
| miR-6723-5p | 1.44 | 1.05-1.96 | 2.17×10^-02^ | 1.49 | 1.08-2.05 | 1.51×10^-02^ |
| miR-505-3p | 2.28 | 1.11-4.66 | 2.47×10^-02^ | 2.18 | 1.06-4.49 | 3.36×10^-02^ |
| miR-24-3p | 2.51 | 1.11-5.66 | 2.68×10^-02^ | 2.44 | 1.07-5.55 | 3.30×10^-02^ |
| miR-4688 | 2.04 | 1.08-3.83 | 2.73×10^-02^ | 2.09 | 1.11-3.96 | 2.32×10^-02^ |
| miR-10a-5p | 2.75 | 1.12-6.78 | 2.80×10^-02^ | 2.67 | 1.08-6.62 | 3.38×10^-02^ |
| miR-561-3p | 0.48 | 0.25-0.93 | 2.92×10^-02^ | 0.51 | 0.27-0.99 | 4.62×10^-02^ |
| miR-6769b-3p | 5.77 | 1.19-27.88 | 2.93×10^-02^ | 5.61 | 1.15-27.46 | 3.34×10^-02^ |
| miR-6727-5p | 0.59 | 0.36-0.97 | 3.62×10^-02^ | 0.57 | 0.35-0.93 | 2.31×10^-02^ |
| miR-4673 | 1.55 | 1.02-2.33 | 3.78×10^-02^ | 1.54 | 1.02-2.33 | 3.90×10^-02^ |
| miR-30d-5p | 3.85 | 1.08-13.74 | 3.79×10^-02^ | 3.94 | 1.11-14.01 | 3.42×10^-02^ |
| miR-324-5p | 1.76 | 1.03-3.00 | 3.97×10^-02^ | 1.76 | 1.02-3.05 | 4.31×10^-02^ |
| miR-744-5p | 1.46 | 1.02-2.10 | 4.01×10^-02^ | 1.50 | 1.03-2.18 | 3.48×10^-02^ |
| miR-6877-5p | 2.25 | 1.04-4.90 | 4.02×10^-02^ | 2.32 | 1.08-4.98 | 3.05×10^-02^ |
| let-7c-5p | 3.76 | 1.05-13.44 | 4.17×10^-02^ | 3.93 | 1.07-14.38 | 3.88×10^-02^ |
| miR-1915-3p | 0.53 | 0.29-0.98 | 4.18×10^-02^ | 0.47 | 0.26-0.86 | 1.37×10^-02^ |
| miR-185-3p | 1.69 | 1.02-2.80 | 4.25×10^-02^ | 1.72 | 1.02-2.91 | 4.37×10^-02^ |
| miR-6789-3p | 2.68 | 1.02-7.02 | 4.51×10^-02^ | 3.05 | 1.11-8.42 | 3.13×10^-02^ |
| miR-6851-3p | 2.10 | 1.02-4.34 | 4.51×10^-02^ | 2.13 | 1.01-4.47 | 4.68×10^-02^ |
| miR-2110 | 2.03 | 1.01-4.08 | 4.73×10^-02^ | 2.08 | 1.01-4.29 | 4.71×10^-02^ |
| miR-6085 | 0.62 | 0.36-1.04 | 7.05×10^-02^ | 0.56 | 0.33-0.95 | 3.09×10^-02^ |
| miR-3940-5p | 0.65 | 4.02-1.06 | 8.43×10^-02^ | 0.60 | 0.37-0.97 | 3.65×10^-02^ |
| miR-107 | 2.41 | 0.98-5.95 | 5.71×10^-02^ | 2.65 | 1.06-6.64 | 3.77×10^-02^ |
| miR-4461 | 1.23 | 0.99-1.53 | 6.20×10^-02^ | 1.26 | 1.01-1.58 | 4.06×10^-02^ |
| miR-1301-5p | 2.67 | 0.93-7.68 | 6.88×10^-02^ | 2.95 | 1.01-8.62 | 4.84×10^-02^ |
| miR-3907 | 2.55 | 0.91-7.15 | 7.52×10^-02^ | 2.86 | 1.00-8.19 | 4.99×10^-02^ |

Note: Model 1 : adjusted for age, sex, cohort. Model 2: adjusted for model1, smoking status, chronic disease, BMI, RBC, WBC, alcohol and education. The table is ranked according to the P values of miRNAs nominally associated with incident lung cancer in model 1. Abbreviations. BMI, body mass index; HR, hazard ratio; RBC, red blood cells; WBC, white blood cells; miRNA, microRNA; CI, confidence interval.

**Table S4. The list of miRNAs nominally associated with incident breast cancer**

| miRNA ID | Model 1 | | | Model 2 | | |
| --- | --- | --- | --- | --- | --- | --- |
|  | HR | 95% CI | *P* value | HR | 95% CI | *P* value |
| miR-3140-3p | 10.07 | 2.44-41.56 | 1.40×10^-03^ | 12.17 | 2.82-52.50 | 8.08×10^-04^ |
| miR-6852-5p | 5.69 | 1.88-17.19 | 2.05×10^-03^ | 5.64 | 1.88-16.90 | 1.99×10^-03^ |
| miR-381-3p | 2.85 | 1.34-6.02 | 6.27×10^-03^ | 2.77 | 1.31-5.85 | 7.58×10^-03^ |
| miR-4291 | 3.11 | 1.36-7.09 | 6.93×10^-03^ | 3.26 | 1.41-7.55 | 5.72×10^-03^ |
| miR-6748-3p | 0.57 | 0.38-0.86 | 7.59×10^-03^ | 0.60 | 0.40-0.91 | 1.68×10^-02^ |
| miR-4758-5p | 0.49 | 0.28-0.88 | 1.75×10^-02^ | 0.50 | 0.28-0.90 | 2.14×10^-02^ |
| miR-6886-3p | 0.66 | 0.46-0.93 | 1.88×10^-02^ | 0.65 | 0.46-0.93 | 1.79×10^-02^ |
| miR-3157-5p | 3.31 | 1.21-9.04 | 1.96×10^-02^ | 3.67 | 1.31-10.22 | 1.31×10^-02^ |
| miR-3152-3p | 4.34 | 1.25-15.09 | 2.08×10^-02^ | 4.43 | 1.25-15.75 | 2.13×10^-02^ |
| miR-6784-5p | 0.58 | 0.36-0.92 | 2.19×10^-02^ | 0.55 | 0.34-0.89 | 1.48×10^-02^ |
| miR-1237-3p | 0.67 | 0.47-0.94 | 2.24×10^-02^ | 0.68 | 0.47-0.97 | 3.11×10^-02^ |
| miR-670-3p | 3.82 | 1.20-12.12 | 2.29×10^-02^ | 3.94 | 1.24-12.49 | 1.98×10^-02^ |
| miR-6821-5p | 0.54 | 0.30-0.94 | 2.97×10^-02^ | 0.50 | 0.28-0.90 | 2.14×10^-02^ |
| miR-3912-5p | 4.33 | 1.11-16.87 | 3.48×10^-02^ | 4.50 | 1.15-17.60 | 3.07×10^-02^ |
| miR-4728-3p | 0.55 | 0.32-0.96 | 3.54×10^-02^ | 0.54 | 0.60-0.94 | 3.05×10^-02^ |
| miR-4676-3p | 2.09 | 1.04-4.22 | 3.89×10^-02^ | 2.11 | 1.04-4.28 | 3.78×10^-02^ |
| miR-4667-5p | 3.33 | 1.06-10.48 | 4.00×10^-02^ | 3.70 | 1.14-11.98 | 2.88×10^-02^ |
| miR-217 | 3.07 | 1.05-8.97 | 4.05×10^-02^ | 3.16 | 1.07-9.31 | 3.74×10^-02^ |
| miR-4421 | 0.73 | 0.53-1.00 | 5.34×10^-02^ | 0.72 | 0.53-0.99 | 4.32×10^-02^ |

Note: Model 1 : adjusted for age, cohort. Model 2: adjusted for model1, smoking status, chronic disease, BMI, RBC, WBC, alcohol and education. The table is ranked according to the P values of miRNAs nominally associated with incident breast cancer in model 1. Abbreviations. BMI, body mass index; HR, hazard ratio; RBC, red blood cells; WBC, white blood cells; miRNA, microRNA; CI, confidence interval.

**Table S5. The list of miRNAs nominally associated with incident colorectal cancer**

| miRNA ID | Model 1 | | | Model 2 | | |
| --- | --- | --- | --- | --- | --- | --- |
|  | HR | 95% CI | *P* value | HR | 95% CI | *P* value |
| miR-6852-5p | 0.46 | 0.28-0.75 | 1.88×10^-03^ | 0.43 | 0.26-0.71 | 8.71×10^-04^ |
| miR-1306-5p | 0.29 | 0.13-0.63 | 2.08×10^-03^ | 0.29 | 0.13-0.66 | 2.90×10^-03^ |
| miR-330-3p | 0.60 | 0.42-0.85 | 4.07×10^-03^ | 0.59 | 0.42-0.84 | 3.30×10^-03^ |
| miR-144-5p | 2.99 | 1.39-6.40 | 4.88×10^-03^ | 3.24 | 1.47-7.14 | 3.48×10^-03^ |
| let-7a-5p | 3.56 | 1.45-8.73 | 5.51×10^-03^ | 3.21 | 1.30-7.90 | 1.13×10^-02^ |
| miR-6821-3p | 0.55 | 0.36-0.84 | 5.73×10^-03^ | 0.59 | 0.39-0.89 | 1.13×10^-02^ |
| miR-4646-5p | 0.50 | 0.30-0.84 | 8.56×10^-03^ | 0.49 | 0.29-0.82 | 6.72×10^-03^ |
| miR-7978 | 0.59 | 0.39-0.90 | 1.44×10^-03^ | 0.55 | 0.36-0.86 | 8.35×10^-03^ |
| miR-3620-3p | 0.64 | 0.44-0.92 | 1.76×10^-02^ | 0.66 | 0.45-0.96 | 2.93×10^-02^ |
| miR-210-3p | 0.19 | 0.05-0.76 | 1.86×10^-02^ | 0.21 | 0.05-0.85 | 2.85×10^-02^ |
| let-7g-5p | 4.55 | 1.29-16.08 | 1.86×10^-02^ | 4.26 | 1.19-15.26 | 2.60×10^-02^ |
| miR-155-5p | 2.47 | 1.16-5.25 | 1.91×10^-02^ | 2.34 | 1.11-4.95 | 2.59×10^-02^ |
| let-7f-5p | 2.50 | 1.10-5.70 | 2.89×10^-02^ | 2.42 | 1.05-5.59 | 2.89×10^-02^ |
| miR-451a | 1.38 | 1.03-1.85 | 2.36×10^-02^ | 1.41 | 1.04-1.92 | 2.64×10^-02^ |
| miR-30b-5p | 3.09 | 1.08-8.89 | 3.61×10^-02^ | 2.92 | 1.01-8.45 | 4.87×10^-02^ |
| miR-764 | 0.59 | 0.35-1.00 | 4.85×10^-02^ | 0.58 | 0.34-0.99 | 4.70×10^-02^ |
| miR-4522 | 0.65 | 0.42-1.00 | 4.87×10^-02^ | 0.65 | 0.43-0.99 | 4.32×10^-02^ |
| miR-5196-5p | 0.44 | 0.19-1.00 | 4.92×10^-02^ | 0.38 | 0.16-0.92 | 3.22×10^-02^ |
| miR-129-5p | 2.00 | 0.98-4.06 | 5.57×10^-02^ | 2.08 | 1.01-4.30 | 4.80×10^-02^ |

Note: Model 1 : adjusted for age, sex, cohort. Model 2: adjusted for model1, smoking status, chronic disease, BMI, RBC, WBC, alcohol and education. The table is ranked according to the P values of miRNAs nominally associated with incident colorectal cancer in model 1. Abbreviations. BMI, body mass index; HR, hazard ratio; RBC, red blood cells; WBC, white blood cells; miRNA, microRNA; CI, confidence interval.

**Table S6. The list of miRNAs nominally associated with incident prostate cancer**

| miRNA ID | Model 1 | | | Model 2 | | |
| --- | --- | --- | --- | --- | --- | --- |
|  | HR | 95% CI | *P* value | HR | 95% CI | *P* value |
| miR-6736-3p | 7.35 | 2.53-21.37 | 2.50×10^-04^ | 7.94 | 2.70-23.40 | 1.71×10^-04^ |
| miR-1307-3p | 2.57 | 1.52-4.32 | 3.93×10^-04^ | 2.66 | 1.58-4.50 | 2.47×10^-04^ |
| miR-1247-3p | 2.72 | 1.52-4.87 | 7.94×10^-04^ | 2.73 | 1.54-4.84 | 6.05×10^-04^ |
| miR-6741-3p | 3.43 | 1.66-7.06 | 8.35×10^-04^ | 3.30 | 1.63-6.68 | 9.02×10^-04^ |
| miR-3943 | 3.29 | 1.61-6.71 | 1.10×10^-03^ | 3.28 | 1.62-6.66 | 1.01×10^-03^ |
| miR-187-3p | 9.60 | 2.38-38.61 | 1.46×10^-03^ | 13.91 | 3.16-61.30 | 5.03×10^-04^ |
| miR-374c-5p | 2.83 | 1.48-5.41 | 1.69×10^-03^ | 2.87 | 1.51-5.47 | 1.35×10^-03^ |
| miR-5694 | 3.82 | 1.65-8.86 | 1.79×10^-03^ | 4.13 | 1.79-9.55 | 8.94×10^-04^ |
| miR-187-5p | 2.44 | 1.37-4.33 | 2.45×10^-03^ | 2.67 | 1.50-4.73 | 7.86×10^-04^ |
| miR-764 | 2.70 | 1.41-5.18 | 2.72×10^-03^ | 2.95 | 1.53-5.69 | 1.24×10^-03^ |
| miR-937-3p | 5.93 | 1.83-19.17 | 2.95×10^-03^ | 6.64 | 1.98-22.28 | 2.16×10^-03^ |
| miR-541-3p | 2.60 | 1.37-4.96 | 3.64×10^-03^ | 2.83 | 1.46-5.48 | 2.01×10^-03^ |
| miR-6870-3p | 2.35 | 1.32-4.18 | 3.65×10^-03^ | 2.49 | 1.36-4.54 | 3.00×10^-03^ |
| miR-6852-3p | 1.79 | 1.19-2.69 | 4.77×10^-03^ | 1.79 | 1.20-2.68 | 4.61×10^-03^ |
| miR-3912-5p | 5.65 | 1.69-18.87 | 4.86×10^-03^ | 6.06 | 1.80-20.38 | 3.60×10^-03^ |
| miR-3687 | 0.40 | 0.21-0.77 | 5.59×10^-03^ | 0.40 | 0.21-0.76 | 5.19×10^-03^ |
| miR-2116-3p | 5.31 | 1.56-18.06 | 7.58×10^-03^ | 6.09 | 1.75-21.26 | 4.57×10^-03^ |
| miR-5587-3p | 2.78 | 1.29-5.99 | 9.28×10^-03^ | 3.18 | 1.43-7.07 | 4.57×10^-03^ |
| miR-3912-3p | 3.90 | 1.38-11.04 | 1.02×10^-02^ | 4.49 | 1.57-12.82 | 4.98×10^-03^ |
| miR-6825-3p | 1.76 | 1.14-2.72 | 1.03×10^-02^ | 1.78 | 1.15-2.74 | 9.10×10^-03^ |
| miR-548j-3p | 3.96 | 1.38-11.41 | 1.06×10^-02^ | 4.80 | 1.58-14.55 | 5.63×10^-03^ |
| miR-2116-5p | 5.03 | 1.46-17.35 | 1.06×10^-02^ | 5.37 | 1.57-18.39 | 7.41×10^-03^ |
| miR-8078 | 0.64 | 0.45-0.90 | 1.08×10^-02^ | 0.61 | 0.42-0.88 | 7.32×10^-03^ |
| miR-3135a | 0.47 | 0.27-0.84 | 1.10×10^-02^ | 0.44 | 0.24-0.80 | 7.20×10^-03^ |
| miR-1233-3p | 1.85 | 1.15-2.99 | 1.11×10^-02^ | 1.85 | 1.16-2.76 | 9.53×10^-03^ |
| miR-3127-3p | 0.58 | 0.38-0.89 | 1.17×10^-02^ | 0.58 | 0.38-0.90 | 1.38×10^-02^ |
| miR-1254 | 0.50 | 0.29-0.86 | 1.18×10^-02^ | 0.49 | 0.28-0.84 | 9.80×10^-03^ |
| miR-561-5p | 2.36 | 1.20-4.66 | 1.30×10^-02^ | 2.44 | 1.24-4.80 | 9.45×10^-03^ |
| miR-4539 | 0.55 | 0.34-0.88 | 1.35×10^-02^ | 0.55 | 0.34-0.89 | 1.44×10^-02^ |
| miR-299-5p | 2.06 | 1.15-3.69 | 1.47×10^-02^ | 2.12 | 1.18-3.81 | 1.19×10^-02^ |
| miR-4746-5p | 2.52 | 1.20-5.30 | 1.47×10^-02^ | 2.63 | 1.26-5.50 | 1.01×10^-02^ |
| miR-4784 | 3.25 | 1.25-8.47 | 1.56×10^-02^ | 3.63 | 1.36-9.66 | 9.83×10^-03^ |
| miR-3613-5p | 3.43 | 1.25-9.44 | 1.69×10^-02^ | 3.84 | 1.34-10.99 | 1.23×10^-02^ |
| miR-6762-3p | 1.80 | 1.10-2.92 | 1.84×10^-02^ | 1.94 | 1.16-3.23 | 1.10×10^-02^ |
| miR-34a-5p | 0.62 | 0.42-0.92 | 1.90×10^-02^ | 0.61 | 0.41-0.91 | 1.58×10^-02^ |
| miR-548ae | 3.05 | 1.18-7.86 | 2.09×10^-02^ | 3.23 | 1.24-8.42 | 1.67×10^-02^ |
| miR-6069 | 1.98 | 1.11-3.55 | 2.14×10^-02^ | 2.01 | 1.13-3.59 | 1.76×10^-02^ |
| miR-4522 | 1.77 | 1.08-2.89 | 2.23×10^-02^ | 1.78 | 1.09-2.91 | 2.15×10^-02^ |
| miR-4507 | 0.69 | 0.50-0.95 | 2.32×10^-02^ | 0.69 | 0.49-0.96 | 2.77×10^-02^ |
| miR-1275 | 0.49 | 0.26-0.91 | 2.49×10^-02^ | 0.49 | 0.26-0.92 | 2.59×10^-02^ |
| miR-4463 | 0.44 | 0.22-0.90 | 2.53×10^-02^ | 0.45 | 0.22-0.93 | 3.00×10^-02^ |
| miR-4667-5p | 2.71 | 1.13-6.50 | 2.54×10^-02^ | 2.76 | 1.14-6.67 | 2.38×10^-02^ |
| miR-1303 | 0.61 | 0.39-0.94 | 2.62×10^-02^ | 0.59 | 0.38-0.92 | 1.95×10^-02^ |
| miR-4753-3p | 2.45 | 1.11-5.41 | 2.73×10^-02^ | 2.50 | 1.12-5.58 | 2.52×10^-02^ |
| miR-566 | 0.67 | 0.47-0.96 | 2.85×10^-02^ | 0.66 | 0.46-0.94 | 2.28×10^-02^ |
| miR-8071 | 0.46 | 0.23-0.92 | 2.86×10^-02^ | 0.43 | 0.21-0.88 | 2.06×10^-02^ |
| miR-141-3p | 2.68 | 1.09-6.59 | 3.12×10^-02^ | 2.80 | 1.13-6.90 | 2.55×10^-02^ |
| miR-556-3p | 2.03 | 1.06-3.86 | 3.18×10^-02^ | 2.05 | 1.07-3.93 | 3.04×10^-02^ |
| miR-143-5p | 2.67 | 1.09-6.55 | 3.19×10^-02^ | 2.80 | 1.14-6.90 | 2.51×10^-02^ |
| miR-6796-3p | 1.81 | 1.05-3.13 | 3.29×10^-02^ | 1.83 | 1.07-3.12 | 2.69×10^-02^ |
| miR-3907 | 2.38 | 1.07-5.30 | 3.32×10^-02^ | 2.33 | 1.05-5.17 | 3.75×10^-02^ |
| miR-3157-5p | 2.43 | 1.06-5.54 | 3.50×10^-02^ | 2.38 | 1.06-5.35 | 3.54×10^-02^ |
| miR-561-3p | 1.68 | 1.03-2.73 | 3.59×10^-02^ | 1.73 | 1.06-2.81 | 2.81×10^-02^ |
| miR-671-5p | 1.54 | 1.03-2.32 | 3.68×10^-02^ | 1.53 | 1.02-2.29 | 3.94×10^-02^ |
| miR-299-3p | 1.74 | 1.03-2.93 | 3.74×10^-02^ | 1.74 | 1.03-2.92 | 3.82×10^-02^ |
| miR-1273c | 0.54 | 0.30-0.97 | 3.79×10^-02^ | 0.53 | 0.29-0.96 | 3.60×10^-02^ |
| miR-4790-5p | 1.67 | 1.03-2.71 | 3.82×10^-02^ | 1.67 | 1.03-2.72 | 3.78×10^-02^ |
| miR-4726-3p | 2.60 | 1.05-6.44 | 3.96×10^-02^ | 2.74 | 1.11-6.77 | 2.91×10^-02^ |
| miR-34b-3p | 2.20 | 1.04-4.69 | 4.00×10^-02^ | 2.36 | 1.10-5.10 | 2.83×10^-02^ |
| miR-6722-5p | 1.70 | 1.02-2.83 | 4.36×10^-02^ | 1.78 | 1.04-3.05 | 3.55×10^-02^ |
| miR-211-3p | 0.73 | 0.54-0.99 | 4.50×10^-02^ | 0.71 | 0.51-0.98 | 3.45×10^-02^ |
| miR-5196-3p | 2.23 | 1.01-4.92 | 4.66×10^-02^ | 2.27 | 1.02-5.05 | 4.51×10^-02^ |
| miR-339-3p | 1.33 | 1.00-1.76 | 4.80×10^-02^ | 1.32 | 1.00-1.74 | 4.92×10^-02^ |
| miR-1273h-5p | 0.70 | 0.49-1.00 | 4.94×10^-02^ | 0.69 | 0.48-0.99 | 4.23×10^-02^ |
| miR-2115-5p | 3.59 | 0.94-13.79 | 6.25×10^-02^ | 4.72 | 1.13-19.70 | 3.33×10^-02^ |
| miR-1207-5p | 1.91 | 0.98-3.72 | 5.59×10^-02^ | 2.08 | 1.05-4.09 | 3.47×10^-02^ |
| miR-2115-3p | 4.31 | 0.96-19.32 | 5.61×10^-02^ | 5.24 | 1.10-24.91 | 3.72×10^-02^ |
| miR-204-3p | 3.15 | 1.00-9.92 | 5.04×10^-02^ | 3.39 | 1.07-10.73 | 3.76×10^-02^ |
| miR-4721 | 1.85 | 0.99-3.44 | 5.21×10^-02^ | 1.99 | 1.04-3.81 | 3.85×10^-02^ |
| miR-8069 | 0.46 | 0.21-1.03 | 5.92×10^-02^ | 0.43 | 0.19-0.96 | 4.05×10^-02^ |
| miR-2276-5p | 1.97 | 0.99-3.93 | 5.45×10^-02^ | 2.12 | 1.03-4.39 | 4.18×10^-02^ |
| miR-1908-5p | 1.68 | 0.98-2.86 | 5.82×10^-02^ | 1.75 | 1.02-3.01 | 4.35×10^-02^ |
| miR-1273e | 0.67 | 0.45-1.01 | 5.41×10^-02^ | 0.66 | 0.44-0.99 | 4.59×10^-02^ |
| miR-6500-3p | 0.79 | 0.61-1.01 | 5.75×10^-02^ | 0.77 | 0.60-1.00 | 4.86×10^-02^ |
| miR-6891-3p | 2.12 | 0.99-4.54 | 5.24×10^-02^ | 2.17 | 1.00-4.67 | 4.87×10^-02^ |
| miR-4727-5p | 1.49 | 0.98-2.28 | 6.37×10^-02^ | 1.55 | 1.00-2.39 | 4.97×10^-02^ |
| miR-4421 | 0.74 | 0.55-1.00 | 5.34×10^-02^ | 0.74 | 0.55-1.00 | 4.98×10^-02^ |

Note: Model 1 : adjusted for age, cohort. Model 2: adjusted for model1, smoking status, chronic disease, BMI, RBC, WBC, alcohol and education. The table is ranked according to the P values of miRNAs nominally associated with incident prostate cancer in model 1. Abbreviations. BMI, body mass index; HR, hazard ratio; RBC, red blood cells; WBC, white blood cells; miRNA, microRNA; CI, confidence interval.

**Table S7. The list of miRNAs nominally associated with incident all causes cancer**

| miRNA ID | Model 1 | | | Model 2 | | |
| --- | --- | --- | --- | --- | --- | --- |
|  | HR | 95% CI | *P* value | HR | 95% CI | *P* value |
| miR-299-3p | 1.36 | 1.11-1.67 | 2.96×10^-03^ | 1.37 | 1.11-1.68 | 3.04×10^-03^ |
| miR-362-5p | 1.33 | 1.08-1.64 | 6.51×10^-03^ | 1.40 | 1.13-1.72 | 1.79×10^-03^ |
| miR-1224-5p | 0.78 | 0.64-0.94 | 1.07×10^-02^ | 0.77 | 0.63-0.93 | 7.28×10^-03^ |
| miR-3907 | 1.46 | 1.08-1.98 | 1.45×10^-02^ | 1.55 | 1.13-2.12 | 6.56×10^-03^ |
| miR-18b-5p | 1.30 | 1.05-1.60 | 1.55×10^-02^ | 1.33 | 1.07-1.65 | 8.99×10^-03^ |
| let-7e-5p | 1.43 | 1.05-1.95 | 2.27×10^-02^ | 1.46 | 1.07-2.00 | 1.63×10^-02^ |
| miR-299-5p | 1.26 | 1.03-1.53 | 2.36×10^-02^ | 1.29 | 1.05-1.57 | 1.44×10^-02^ |
| miR-1231 | 0.81 | 0.68-0.98 | 2.71×10^-02^ | 0.78 | 0.63-0.93 | 8.05×10^-03^ |
| miR-4436a | 0.73 | 0.55-0.98 | 3.28×10^-02^ | 0.75 | 0.56-1.00 | 4.86×10^-02^ |
| miR-4676-3p | 1.23 | 1.01-1.51 | 4.31×10^-02^ | 1.50 | 1.04-2.15 | 3.01×10^-02^ |
| miR-6787-5p | 1.17 | 1.00-1.37 | 4.40×10^-02^ | 1.19 | 1.02-1.39 | 2.52×10^-02^ |
| miR-4667-5p | 1.45 | 1.01-2.08 | 4.43×10^-02^ | 1.23 | 1.01-1.51 | 3.01×10^-02^ |
| miR-4784 | 1.40 | 1.00-1.96 | 4.69×10^-02^ | 1.42 | 1.01-1.83 | 4.42×10^-02^ |
| miR-3140-3p | 1.48 | 0.94-2.34 | 9.11×10^-02^ | 1.71 | 1.07-2.72 | 2.43×10^-02^ |
| let-7a-5p | 1.30 | 0.96-1.77 | 9.16×10^-02^ | 1.41 | 1.04-1.93 | 2.94×10^-02^ |
| miR-10a-5p | 1.32 | 0.99-1.77 | 6.25×10^-02^ | 1.36 | 1.01-1.83 | 4.44×10^-02^ |
| miR-4449 | 0.91 | 0.73-1.14 | 4.29×10^-01^ | 0.79 | 0.62-1.00 | 4.67×10^-02^ |
| miR-139-5p | 1.41 | 0.96-2.07 | 8.14×10^-02^ | 1.49 | 1.00-2.20 | 4.72×10^-02^ |
| miR-4486 | 0.72 | 0.46-1.15 | 1.74×10^-01^ | 0.63 | 0.40-0.99 | 4.74×10^-02^ |

Note: Model 1 : adjusted for age, sex, cohort. Model 2: adjusted for model1, smoking status, chronic disease, BMI, RBC, WBC, alcohol and education. The table is ranked according to the P values of miRNAs nominally associated with incident pan cancer in model 1. Abbreviations. BMI, body mass index; HR, hazard ratio; RBC, red blood cells; WBC, white blood cells; miRNA, microRNA; CI, confidence interval.

**Table S8. Two miRNAs nominally associated with incidence of three different cancer types**

| miRNAs ID | Rotterdam Study | | | | | Reported in previous studies |
| --- | --- | --- | --- | --- | --- | --- |
| Incident cancer | Blood | Lung | Breast | Colorectal | Prostate | Any cancer |
| miR-3157-5p | ✓ | - | ✓ | - | ✓ | Breast[15]; Blood[16] |
| miR-3912-5p | ✓ | - | ✓ | - | ✓ | Liver[17]; CNS[18]; Thyroid[19] |

Note: The nominal association threshold is *p* < 0.05. Abbreviations: CNS, central nervous system; miRNA, microRNA.

**Table S9. The list of miRNAs nominally associated with prevalent hematological tumors**

| miRNA ID | Model 1 | | | Model 2 | | | |  |
| --- | --- | --- | --- | --- | --- | --- | --- | --- |
|  | Beta | SE | *P* value | | Beta | SE | *P* value | |
| miR-4433b-5p | -2.30 | 0.42 | 3.25×10^-08^ | | -2.22 | 0.45 | 8.47×10^-07*^ | |
| **miR-7111-5p** | **1.62** | **0.29** | **3.27×10^-08^** | | **1.56** | **0.31** | **5.85×10^-07*^** | |
| miR-1234-3p | -4.83 | 0.94 | 3.20×10^-07^ | | -4.54 | 0.98 | 3.28×10^-06*^ | |
| miR-4655-3p | -3.47 | 0.68 | 3.90×10^-07^ | | -3.08 | 0.73 | 2.65×10^-05*^ | |
| miR-6771-3p | -4.51 | 0.89 | 3.92×10^-07^ | | -4.15 | 0.93 | 8.06×10^-06*^ | |
| miR-4274 | -5.04 | 1.02 | 6.92×10^-07^ | | -4.86 | 1.14 | 2.11×10^-05*^ | |
| miR-187-3p | -3.13 | 0.63 | 7.80×10^-07^ | | -2.81 | 0.70 | 6.06×10^-05*^ | |
| miR-6799-5p | 1.49 | 0.30 | 8.10×10^-07^ | | 1.73 | 0.38 | 4.22×10^-06*^ | |
| miR-4728-5p | 1.82 | 0.37 | 8.68×10^-07^ | | 1.79 | 0.40 | 8.06×10^-06*^ | |
| miR-1275 | 1.78 | 0.36 | 8.85×10^-07^ | | 1.89 | 0.41 | 3.83×10^-06*^ | |
| miR-504-3p | 1.93 | 0.40 | 1.03×10^-06^ | | 1.82 | 0.41 | 1.07×10^-05*^ | |
| miR-6086 | 1.60 | 0.33 | 1.06×10^-06^ | | 1.60 | 0.37 | 1.25×10^-05*^ | |
| miR-6880-5p | 2.64 | 0.54 | 1.22×10^-06^ | | 2.73 | 0.62 | 1.13×10^-05*^ | |
| **miR-6124** | **1.15** | **0.24** | **1.26×10^-06^** | | **1.12** | **0.27** | **3.15×10^-05*^** | |
| miR-155-5p | 2.48 | 0.51 | 1.39×10^-06^ | | 2.26 | 0.53 | 2.24×10^-05*^ | |
| miR-6797-5p | 1.84 | 0.39 | 2.04×10^-06^ | | 1.82 | 0.42 | 1.86×10^-05*^ | |
| **miR-6870-3p** | **-1.35** | **0.29** | **2.33×10^-06^** | | **-1.33** | **0.30** | **9.99×10^-06*^** | |
| miR-6870-5p | 1.01 | 0.22 | 2.50×10^-06^ | | 0.91 | 0.22 | 4.75×10^-05*^ | |
| miR-7107-5p | 1.68 | 0.36 | 2.65×10^-06^ | | 1.78 | 0.41 | 1.54×10^-05*^ | |
| **miR-4478** | **1.60** | **0.34** | **2.73×10^-06^** | | **1.69** | **0.40** | **2.17×10^-05*^** | |
| miR-7150 | 1.61 | 0.34 | 3.01×10^-06^ | | 1.75 | 0.40 | 1.38×10^-05*^ | |
| miR-6894-5p | 1.64 | 0.36 | 4.89×10^-06^ | | 1.63 | 0.41 | 8.43×10^-05*^ | |
| **miR-4430** | **1.64** | **0.36** | **5.21×10^-06^** | | **1.75** | **0.41** | **1.75×10^-05*^** | |
| miR-126-3p | -1.81 | 0.40 | 5.73×10^-06^ | | -1.76 | 0.45 | 8.69×10^-05^ | |
| miR-4800-5p | 2.83 | 0.63 | 6.11×10^-06^ | | 2.86 | 0.68 | 2.45×10^-05*^ | |
| miR-4656 | 1.88 | 0.42 | 6.70×10^-06^ | | 1.77 | 0.43 | 4.42×10^-05*^ | |
| miR-6780b-5p | 1.44 | 0.32 | 6.78×10^-06^ | | 1.49 | 0.35 | 2.01×10^-05*^ | |
| **miR-6778-5p** | **1.91** | **0.42** | **7.21×10^-06^** | | **2.00** | **0.49** | **3.76×10^-05*^** | |
| miR-2115-3p | -2.25 | 0.50 | 7.22×10^-06^ | | -1.98 | 0.57 | 4.74×10^-04^ | |
| miR-6812-5p | 3.11 | 0.70 | 7.95×10^-06^ | | 3.07 | 0.73 | 2.91×10^-05*^ | |
| miR-29c-5p | 2.71 | 0.61 | 8.48×10^-06^ | | 2.53 | 0.62 | 4.63×10^-05*^ | |
| miR-3648 | 1.21 | 0.27 | 9.63×10^-06^ | | 1.33 | 0.31 | 1.93×10^-05*^ | |
| miR-765 | 1.95 | 0.44 | 9.94×10^-06^ | | 1.88 | 0.49 | 1.13×10^-04^ | |
| miR-1202 | -4.05 | 0.92 | 9.98×10^-06^ | | -3.64 | 0.93 | 8.39×10^-05*^ | |
| miR-7106-5p | 1.94 | 0.44 | 1.12×10^-05^ | | 1.91 | 0.49 | 9.54×10^-05^ | |
| miR-654-5p | **1.29** | **0.29** | **1.15×10^-05^** | | **1.31** | **0.31** | **2.22×10^-05*^** | |
| miR-6127 | 2.38 | 0.54 | 1.27×10^-05^ | | 2.47 | 0.61 | 5.84×10^-05*^ | |
| miR-33b-5p | -3.12 | 0.72 | 1.42×10^-05^ | | -2.84 | 0.76 | 1.74×10^-04^ | |
| miR-6771-5p | 2.13 | 0.49 | 1.46×10^-05^ | | 2.02 | 0.53 | 1.41×10^-04^ | |
| miR-5703 | 1.73 | 0.41 | 2.01×10^-05^ | | 1.71 | 0.45 | 1.27×10^-04^ | |
| miR-4309 | -2.61 | 0.61 | 2.21×10^-05^ | | -2.31 | 0.67 | 6.17×10^-04^ | |
| miR-4270 | 1.79 | 0.42 | 2.30×10^-05^ | | 1.75 | 0.46 | 1.57×10^-04^ | |
| miR-361-3p | 2.69 | 0.64 | 2.54×10^-05^ | | 2.64 | 0.69 | 1.19×10^-04^ | |
| miR-3184-3p | -1.37 | 0.33 | 2.69×10^-05^ | | -1.31 | 0.34 | 1.36×10^-04^ | |
| miR-3140-5p | -2.78 | 0.66 | 2.90×10^-05^ | | -2.53 | 0.74 | 6.35×10^-04^ | |
| miR-2115-5p | -2.34 | 0.56 | 3.02×10^-05^ | | -2.00 | 0.62 | 1.23×10^-03^ | |
| miR-6785-3p | -3.97 | 0.96 | 3.44×10^-05^ | | -3.83 | 0.97 | 7.94×10^-05*^ | |
| **miR-5196-5p** | **1.23** | **0.30** | **3.49×10^-05^** | | **1.14** | **0.30** | **1.48×10^-04^** | |
| miR-4449 | 1.03 | 0.25 | 3.62×10^-05^ | | 1.11 | 0.27 | 5.79×10^-05*^ | |
| **miR-4534** | **1.57** | **0.38** | **3.69×10^-05^** | | **1.47** | **0.42** | **4.98×10^-04^** | |
| miR-3162-5p | 1.78 | 0.43 | 3.73×10^-05^ | | 1.77 | 0.45 | 7.92×10^-05*^ | |
| miR-34a-5p | 1.89 | 0.46 | 4.19×10^-05^ | | 2.07 | 0.48 | 1.60×10^-05*^ | |
| miR-29c-3p | 2.61 | 0.64 | 4.34×10^-05^ | | 2.36 | 0.63 | 1.81×10^-04^ | |
| miR-4291 | -2.18 | 0.54 | 5.04×10^-05^ | | -1.95 | 0.58 | 7.88×10^-04^ | |
| miR-3912-3p | -2.05 | 0.51 | 5.32×10^-05^ | | -1.85 | 0.56 | 9.53×10^-04^ | |
| miR-6736-3p | -1.81 | 0.45 | 5.62×10^-05^ | | -1.98 | 0.49 | 5.81×10^-05*^ | |
| **miR-4644** | **1.11** | **0.28** | **5.87×10^-05^** | | **0.95** | **0.28** | **6.89×10^-04^** | |
| miR-6742-5p | -2.53 | 0.63 | 5.89×10^-05^ | | -2.28 | 0.65 | 4.91×10^-04^ | |
| miR-3140-3p | -2.64 | 0.66 | 6.15×10^-05^ | | -2.31 | 0.72 | 1.32×10^-03^ | |
| miR-6803-5p | 1.79 | 0.45 | 6.25×10^-05^ | | 2.04 | 0.53 | 1.32×10^-04^ | |
| miR-6804-3p | -2.99 | 0.75 | 6.67×10^-05^ | | -2.67 | 0.76 | 4.58×10^-04^ | |
| miR-3180-3p | -1.91 | 0.48 | 6.97×10^-05^ | | -1.67 | 0.47 | 4.42×10^-04^ | |
| miR-4640-3p | 2.17 | 0.55 | 7.45×10^-05^ | | 2.36 | 0.59 | 7.42×10^-05*^ | |
| miR-6088 | 0.94 | 0.24 | 7.80×10^-05^ | | 1.12 | 0.27 | 2.66×10^-05*^ | |
| miR-640 | -2.10 | 0.53 | 8.60×10^-05^ | | -2.00 | 0.56 | 3.78×10^-04^ | |
| miR-3940-5p | 1.23 | 0.31 | 8.71×10^-05^ | | 1.28 | 0.32 | 7.99×10^-05*^ | |
| miR-187-5p | -2.56 | 0.65 | 9.26×10^-05^ | | -2.33 | 0.74 | 1.73×10^-03^ | |
| miR-6821-5p | 1.84 | 0.47 | 9.51×10^-05^ | | 1.93 | 0.48 | 6.39×10^-05*^ | |
| miR-3180 | -1.79 | 0.46 | 1.02×10^-04^ | | -1.57 | 0.45 | 5.33×10^-04^ | |
| miR-6845-5p | 1.47 | 0.38 | 1.03×10^-04^ | | 1.35 | 0.40 | 6.33×10^-04^ | |
| miR-6794-3p | -2.56 | 0.66 | 1.04×10^-04^ | | -2.09 | 0.69 | 2.57×10^-03^ | |
| miR-181c-5p | -2.37 | 0.61 | 1.08×10^-04^ | | -2.13 | 0.66 | 1.36×10^-03^ | |
| miR-34b-3p | -2.51 | 0.65 | 1.14×10^-04^ | | -2.27 | 0.68 | 8.66×10^-04^ | |
| miR-212-3p | -2.41 | 0.63 | 1.23×10^-04^ | | -1.99 | 0.68 | 3.55×10^-03^ | |
| miR-4257 | -2.42 | 0.63 | 1.27×10^-04^ | | -2.13 | 0.65 | 1.12×10^-03^ | |
| miR-6789-5p | -2.03 | 0.53 | 1.31×10^-04^ | | -1.92 | 0.54 | 3.44×10^-04^ | |
| miR-658 | -1.93 | 0.51 | 1.49×10^-04^ | | -1.79 | 0.51 | 4.04×10^-04^ | |
| miR-6729-3p | -3.56 | 0.94 | 1.53×10^-04^ | | -3.32 | 0.95 | 4.74×10^-04^ | |
| miR-6165 | -1.62 | 0.43 | 1.55×10^-04^ | | -1.46 | 0.41 | 3.65×10^-04^ | |
| miR-6775-3p | -3.40 | 0.90 | 1.64×10^-04^ | | -2.87 | 0.90 | 1.42×10^-03^ | |
| miR-222-3p | 2.75 | 0.73 | 1.66×10^-04^ | | 2.68 | 0.71 | 1.71×10^-04^ | |
| miR-6798-5p | 1.24 | 0.33 | 1.70×10^-04^ | | 1.29 | 0.33 | 1.08×10^-04^ | |
| miR-3912-5p | -2.07 | 0.55 | 1.72×10^-04^ | | -1.78 | 0.62 | 3.90×10^-03^ | |
| miR-3157-3p | -1.98 | 0.53 | 1.91×10^-04^ | | -1.92 | 0.59 | 1.12×10^-03^ | |
| miR-6769b-3p | -3.25 | 0.87 | 1.98×10^-04^ | | -2.75 | 0.91 | 2.41×10^-03^ | |
| miR-6809-5p | -2.21 | 0.60 | 2.21×10^-04^ | | -1.97 | 0.63 | 1.78×10^-03^ | |
| miR-6775-5p | 1.14 | 0.31 | 2.25×10^-04^ | | 1.09 | 0.32 | 5.24×10^-04^ | |
| miR-1249 | -2.22 | 0.60 | 2.31×10^-04^ | | -2.34 | 0.65 | 3.32×10^-04^ | |
| miR-5739 | -1.17 | 0.32 | 2.43×10^-04^ | | -1.08 | 0.31 | 6.12×10^-04^ | |
| miR-4279 | -2.02 | 0.56 | 2.75×10^-04^ | | -1.73 | 0.61 | 4.77×10^-03^ | |
| miR-6873-3p | -1.12 | 0.31 | 2.77×10^-04^ | | -0.95 | 0.32 | 3.42×10^-03^ | |
| miR-210-5p | -3.84 | 1.06 | 3.12×10^-04^ | | -3.37 | 1.06 | 1.44×10^-03^ | |
| miR-2116-3p | -1.96 | 0.54 | 3.13×10^-04^ | | -2.06 | 0.57 | 3.27×10^-04^ | |
| miR-1247-3p | -1.32 | 0.37 | 3.30×10^-04^ | | -1.28 | 0.38 | 7.81×10^-04^ | |
| miR-3157-5p | -2.07 | 0.58 | 3.36×10^-04^ | | -1.81 | 0.62 | 3.54×10^-03^ | |
| miR-4463 | 1.60 | 0.45 | 3.43×10^-04^ | | 1.65 | 0.48 | 6.62×10^-04^ | |
| **miR-4292** | **2.14** | **0.60** | **3.48×10^-04^** | | **2.20** | **0.64** | **5.90×10^-04^** | |
| miR-5001-5p | -3.26 | 0.91 | 3.54×10^-04^ | | -2.58 | 0.92 | 5.28×10^-03^ | |
| miR-143-5p | -1.71 | 0.48 | 3.92×10^-04^ | | -1.94 | 0.53 | 2.85×10^-04^ | |
| miR-497-3p | -1.92 | 0.54 | 3.95×10^-04^ | | -1.75 | 0.64 | 6.05×10^-03^ | |
| miR-29b-3p | 2.66 | 0.76 | 4.32×10^-04^ | | 2.66 | 0.79 | 7.62×10^-04^ | |
| miR-6813-3p | -3.09 | 0.88 | 4.33×10^-04^ | | -2.96 | 0.89 | 9.14×10^-04^ | |
| miR-5694 | -1.81 | 0.51 | 4.37×10^-04^ | | -1.76 | 0.53 | 9.66×10^-04^ | |
| miR-197-5p | -1.09 | 0.31 | 4.40×10^-04^ | | -1.05 | 0.32 | 8.35×10^-04^ | |
| miR-1307-3p | -1.42 | 0.41 | 4.58×10^-04^ | | -1.29 | 0.40 | 1.36×10^-03^ | |
| miR-3652 | 1.05 | 0.30 | 4.60×10^-04^ | | 1.02 | 0.32 | 1.66×10^-03^ | |
| miR-648 | -2.09 | 0.60 | 4.62×10^-04^ | | -1.80 | 0.67 | 7.35×10^-03^ | |
| miR-3689d | -1.42 | 0.41 | 5.53×10^-04^ | | -1.49 | 0.42 | 4.49×10^-04^ | |
| miR-6760-3p | -1.95 | 0.57 | 5.73×10^-04^ | | -1.84 | 0.59 | 1.86×10^-03^ | |
| miR-2116-5p | -1.56 | 0.46 | 6.09×10^-04^ | | -1.39 | 0.50 | 5.55×10^-03^ | |
| miR-320c | -2.23 | 0.65 | 6.35×10^-04^ | | -2.25 | 0.66 | 6.03×10^-04^ | |
| miR-210-3p | -3.44 | 1.01 | 6.37×10^-04^ | | -3.02 | 1.08 | 5.22×10^-03^ | |
| miR-320b | -2.12 | 0.63 | 7.23×10^-04^ | | -2.21 | 0.64 | 5.74×10^-04^ | |
| miR-4795-5p | -2.03 | 0.61 | 8.18×10^-04^ | | -1.69 | 0.64 | 7.80×10^-03^ | |
| miR-149-3p | 1.00 | 0.30 | 8.21×10^-04^ | | 1.19 | 0.33 | 2.69×10^-04^ | |
| miR-1290 | 1.07 | 0.32 | 8.31×10^-04^ | | 1.02 | 0.33 | 1.78×10^-03^ | |
| miR-7851-3p | 1.33 | 0.40 | 8.93×10^-04^ | | 1.41 | 0.43 | 1.14×10^-03^ | |
| miR-27a-3p | -1.64 | 0.49 | 8.98×10^-04^ | | -1.51 | 0.55 | 5.91×10^-03^ | |
| miR-937-3p | -1.79 | 0.54 | 9.31×10^-04^ | | -1.56 | 0.57 | 6.37×10^-03^ | |
| miR-1244 | 1.61 | 0.49 | 9.77×10^-04^ | | 1.56 | 0.53 | 3.32×10^-03^ | |
| miR-6892-3p | -2.17 | 0.66 | 1.01×10^-03^ | | -2.33 | 0.68 | 5.98×10^-04^ | |
| miR-1273g-5p | 2.04 | 0.62 | 1.03×10^-03^ | | 1.95 | 0.68 | 4.11×10^-03^ | |
| miR-6728-3p | -1.57 | 0.48 | 1.11×10^-03^ | | -1.58 | 0.50 | 1.49×10^-03^ | |
| miR-4459 | 1.47 | 0.45 | 1.13×10^-03^ | | 1.42 | 0.49 | 3.78×10^-03^ | |
| miR-6715b-3p | -1.64 | 0.51 | 1.22×10^-03^ | | -1.44 | 0.54 | 7.75×10^-03^ | |
| miR-7114-3p | -2.25 | 0.70 | 1.24×10^-03^ | | -1.96 | 0.69 | 4.60×10^-03^ | |
| miR-6782-5p | 0.92 | 0.28 | 1.27×10^-03^ | | 0.95 | 0.30 | 1.73×10^-03^ | |
| miR-29a-3p | 1.99 | 0.62 | 1.29×10^-03^ | | 1.85 | 0.63 | 3.18×10^-03^ | |
| **miR-1915-3p** | **1.09** | **0.34** | **1.32×10^-03^** | | **1.12** | **0.35** | **1.25×10^-03^** | |
| miR-146a-3p | -2.39 | 0.74 | 1.32×10^-03^ | | -2.08 | 0.76 | 6.02×10^-03^ | |
| miR-4745-3p | -1.55 | 0.49 | 1.41×10^-03^ | | -1.23 | 0.53 | 2.01×10^-02^ | |
| miR-28-5p | 2.48 | 0.78 | 1.44×10^-03^ | | 2.53 | 0.77 | 1.05×10^-03^ | |
| miR-320a | -1.77 | 0.56 | 1.48×10^-03^ | | -1.78 | 0.56 | 1.38×10^-03^ | |
| miR-548ak | -1.78 | 0.56 | 1.49×10^-03^ | | -1.61 | 0.59 | 6.64×10^-03^ | |
| miR-6727-5p | 1.29 | 0.41 | 1.51×10^-03^ | | 1.35 | 0.41 | 1.01×10^-03^ | |
| miR-5587-3p | -1.83 | 0.58 | 1.51×10^-03^ | | -1.59 | 0.60 | 7.86×10^-03^ | |
| miR-1343-5p | 1.17 | 0.37 | 1.58×10^-03^ | | 1.26 | 0.44 | 3.80×10^-03^ | |
| miR-6738-5p | 1.41 | 0.45 | 1.61×10^-03^ | | 1.53 | 0.48 | 1.49×10^-03^ | |
| miR-16-5p | -1.50 | 0.48 | 1.71×10^-03^ | | -1.41 | 0.57 | 1.31×10^-02^ | |
| miR-6794-5p | 3.16 | 1.01 | 1.74×10^-03^ | | 3.30 | 1.17 | 4.66×10^-03^ | |
| miR-4758-5p | 1.81 | 0.58 | 1.74×10^-03^ | | 1.79 | 0.66 | 6.43×10^-03^ | |
| miR-374b-3p | -1.94 | 0.62 | 1.75×10^-03^ | | -1.62 | 0.70 | 1.98×10^-02^ | |
| miR-561-5p | -1.74 | 0.56 | 1.81×10^-03^ | | -1.46 | 0.61 | 1.75×10^-02^ | |
| miR-4753-5p | -1.84 | 0.60 | 2.08×10^-03^ | | -1.54 | 0.64 | 1.56×10^-02^ | |
| miR-7845-5p | 1.89 | 0.62 | 2.22×10^-03^ | | 1.80 | 0.61 | 3.14×10^-03^ | |
| let-7i-5p | 2.63 | 0.86 | 2.22×10^-03^ | | 2.71 | 0.85 | 1.40×10^-03^ | |
| miR-4795-3p | -1.53 | 0.50 | 2.32×10^-03^ | | -1.22 | 0.51 | 1.74×10^-02^ | |
| miR-4664-3p | -1.46 | 0.48 | 2.37×10^-03^ | | -1.43 | 0.51 | 5.65×10^-03^ | |
| miR-874-3p | -1.86 | 0.61 | 2.42×10^-03^ | | -1.97 | 0.65 | 2.26×10^-03^ | |
| miR-3937 | -0.96 | 0.32 | 2.48×10^-03^ | | -0.91 | 0.31 | 3.95×10^-03^ | |
| miR-1287-5p | -1.13 | 0.38 | 2.65×10^-03^ | | -1.10 | 0.37 | 2.93×10^-03^ | |
| miR-6741-5p | 0.66 | 0.22 | 2.73×10^-03^ | | 0.76 | 0.23 | 1.26×10^-03^ | |
| miR-625-5p | 1.79 | 0.60 | 2.99×10^-03^ | | 1.84 | 0.62 | 3.02×10^-03^ | |
| miR-1307-5p | -1.77 | 0.60 | 3.18×10^-03^ | | -1.62 | 0.57 | 4.69×10^-03^ | |
| miR-34c-5p | -1.31 | 0.45 | 3.23×10^-03^ | | -1.21 | 0.47 | 9.63×10^-03^ | |
| miR-764 | -1.18 | 0.40 | 3.34×10^-03^ | | -1.17 | 0.42 | 5.11×10^-03^ | |
| miR-217 | -1.49 | 0.51 | 3.53×10^-03^ | | -1.24 | 0.53 | 1.85×10^-02^ | |
| miR-181a-5p | -1.91 | 0.66 | 4.03×10^-03^ | | -1.61 | 0.71 | 2.35×10^-02^ | |
| miR-133a-5p | -1.47 | 0.51 | 4.16×10^-03^ | | -1.27 | 0.55 | 1.98×10^-02^ | |
| miR-670-3p | -1.61 | 0.56 | 4.24×10^-03^ | | -1.33 | 0.58 | 2.26×10^-02^ | |
| miR-2276-3p | -0.82 | 0.29 | 4.29×10^-03^ | | -0.79 | 0.29 | 7.30×10^-03^ | |
| miR-4793-5p | -1.15 | 0.41 | 4.86×10^-03^ | | -1.12 | 0.44 | 1.04×10^-02^ | |
| miR-548ae | -1.44 | 0.52 | 5.40×10^-03^ | | -1.30 | 0.53 | 1.44×10^-02^ | |
| miR-6085 | 1.02 | 0.37 | 6.05×10^-03^ | | 1.12 | 0.39 | 3.75×10^-03^ | |
| miR-6800-3p | -1.49 | 0.54 | 6.06×10^-03^ | | -1.61 | 0.58 | 5.52×10^-03^ | |
| miR-4707-3p | 1.73 | 0.63 | 6.08×10^-03^ | | 1.48 | 0.64 | 2.05×10^-02^ | |
| miR-7108-5p | 1.52 | 0.55 | 6.22×10^-03^ | | 1.39 | 0.56 | 1.42×10^-02^ | |
| miR-126-5p | -0.97 | 0.36 | 6.25×10^-03^ | | -0.83 | 0.41 | 4.16×10^-02^ | |
| miR-6750-5p | -0.83 | 0.31 | 6.48×10^-03^ | | -0.77 | 0.31 | 1.18×10^-02^ | |
| miR-320d | -1.89 | 0.70 | 6.81×10^-03^ | | -2.01 | 0.71 | 4.42×10^-03^ | |
| miR-22-3p | -1.11 | 0.42 | 7.60×10^-03^ | | -1.04 | 0.43 | 1.59×10^-02^ | |
| miR-204-3p | -2.49 | 0.96 | 9.19×10^-03^ | | -2.40 | 0.95 | 1.15×10^-02^ | |
| miR-4681 | -0.76 | 0.29 | 9.31×10^-03^ | | -0.80 | 0.33 | 1.44×10^-02^ | |
| miR-23a-3p | -1.59 | 0.61 | 9.50×10^-03^ | | -1.51 | 0.65 | 2.12×10^-02^ | |
| miR-6784-5p | 1.53 | 0.59 | 1.00×10^-02^ | | 1.55 | 0.64 | 1.60×10^-02^ | |
| miR-1233-3p | -1.39 | 0.54 | 1.04×10^-02^ | | -1.42 | 0.52 | 6.85×10^-03^ | |
| miR-6802-5p | -1.30 | 0.51 | 1.05×10^-02^ | | -1.14 | 0.50 | 2.36×10^-02^ | |
| miR-3155b | -1.60 | 0.62 | 1.06×10^-02^ | | -1.69 | 0.66 | 1.10×10^-02^ | |
| miR-6819-3p | -2.81 | 1.11 | 1.09×10^-02^ | | -2.71 | 1.05 | 1.02×10^-02^ | |
| miR-30e-5p | 2.36 | 0.93 | 1.16×10^-02^ | | 2.29 | 0.97 | 1.77×10^-02^ | |
| miR-23a-5p | 1.51 | 0.61 | 1.26×10^-02^ | | 1.50 | 0.63 | 1.77×10^-02^ | |
| miR-451a | -0.73 | 0.29 | 1.29×10^-02^ | | -0.56 | 0.32 | 8.39×10^-02^ | |
| miR-561-3p | -1.32 | 0.53 | 1.32×10^-02^ | | -1.08 | 0.52 | 3.76×10^-02^ | |
| miR-1306-5p | -1.78 | 0.72 | 1.32×10^-02^ | | -1.84 | 0.71 | 9.69×10^-03^ | |
| miR-5006-5p | -2.07 | 0.85 | 1.46×10^-02^ | | -1.93 | 0.83 | 2.05×10^-02^ | |
| miR-2861 | 0.85 | 0.35 | 1.47×10^-02^ | | 0.64 | 0.37 | 8.85×10^-02^ | |
| miR-6796-3p | -1.38 | 0.57 | 1.49×10^-02^ | | -1.40 | 0.55 | 1.19×10^-02^ | |
| miR-23b-3p | -1.57 | 0.66 | 1.76×10^-02^ | | -1.29 | 0.69 | 6.21×10^-02^ | |
| miR-4535 | -1.77 | 0.75 | 1.77×10^-02^ | | -1.54 | 0.73 | 3.46×10^-02^ | |
| miR-1207-5p | -1.70 | 0.72 | 1.80×10^-02^ | | -1.82 | 0.70 | 9.30×10^-03^ | |
| miR-662 | -1.01 | 0.43 | 1.81×10^-02^ | | -1.15 | 0.48 | 1.73×10^-02^ | |
| miR-10b-5p | -0.91 | 0.39 | 1.90×10^-02^ | | -0.73 | 0.48 | 1.25×10^-01^ | |
| miR-500b-3p | -1.23 | 0.54 | 2.11×10^-02^ | | -1.50 | 0.59 | 1.11×10^-02^ | |
| miR-3141 | 1.09 | 0.48 | 2.15×10^-02^ | | 0.93 | 0.49 | 5.80×10^-02^ | |
| miR-1224-5p | 1.13 | 0.49 | 2.21×10^-02^ | | 1.08 | 0.51 | 3.24×10^-02^ | |
| miR-1228-3p | -0.70 | 0.31 | 2.21×10^-02^ | | -0.82 | 0.32 | 1.14×10^-02^ | |
| miR-4429 | -1.61 | 0.70 | 2.24×10^-02^ | | -1.73 | 0.72 | 1.60×10^-02^ | |
| let-7g-5p | 2.39 | 1.05 | 2.30×10^-02^ | | 2.66 | 1.03 | 9.74×10^-03^ | |
| miR-877-3p | -1.45 | 0.64 | 2.34×10^-02^ | | -1.23 | 0.62 | 4.73×10^-02^ | |
| miR-1237-5p | 1.05 | 0.47 | 2.40×10^-02^ | | 1.07 | 0.48 | 2.54×10^-02^ | |
| miR-541-3p | -0.57 | 0.26 | 2.50×10^-02^ | | -0.68 | 0.28 | 1.49×10^-02^ | |
| miR-6789-3p | -0.96 | 0.43 | 2.52×10^-02^ | | -1.09 | 0.44 | 1.40×10^-02^ | |
| miR-6511a-5p | -1.91 | 0.86 | 2.63×10^-02^ | | -2.19 | 0.86 | 1.11×10^-02^ | |
| miR-556-5p | -0.85 | 0.39 | 2.68×10^-02^ | | -0.90 | 0.42 | 3.27×10^-02^ | |
| miR-4727-5p | -0.64 | 0.29 | 2.74×10^-02^ | | -0.70 | 0.31 | 2.48×10^-02^ | |
| miR-342-3p | 1.54 | 0.70 | 2.85×10^-02^ | | 1.76 | 0.67 | 8.73×10^-03^ | |
| miR-6869-5p | 1.63 | 0.75 | 2.95×10^-02^ | | 1.72 | 0.75 | 2.12×10^-02^ | |
| miR-4690-5p | 1.08 | 0.50 | 3.01×10^-02^ | | 1.05 | 0.51 | 3.99×10^-02^ | |
| miR-6726-5p | 1.44 | 0.67 | 3.18×10^-02^ | | 1.43 | 0.70 | 4.15×10^-02^ | |
| miR-4695-5p | 0.72 | 0.34 | 3.22×10^-02^ | | 0.70 | 0.36 | 5.44×10^-02^ | |
| miR-10a-5p | -1.05 | 0.49 | 3.35×10^-02^ | | -0.70 | 0.53 | 1.87×10^-01^ | |
| miR-4713-3p | -0.71 | 0.34 | 3.37×10^-02^ | | -0.63 | 0.35 | 6.67×10^-02^ | |
| miR-4646-5p | 1.49 | 0.71 | 3.45×10^-02^ | | 1.52 | 0.75 | 4.31×10^-02^ | |
| miR-150-5p | 1.16 | 0.55 | 3.51×10^-02^ | | 1.12 | 0.49 | 2.37×10^-02^ | |
| miR-532-3p | 1.36 | 0.65 | 3.71×10^-02^ | | 1.31 | 0.69 | 5.58×10^-02^ | |
| miR-6791-3p | -0.51 | 0.25 | 3.72×10^-02^ | | -0.48 | 0.25 | 4.92×10^-02^ | |
| miR-4798-3p | -0.91 | 0.44 | 3.73×10^-02^ | | -0.83 | 0.45 | 6.51×10^-02^ | |
| miR-2392 | 1.16 | 0.56 | 3.83×10^-02^ | | 1.23 | 0.59 | 3.66×10^-02^ | |
| miR-3943 | -0.98 | 0.47 | 3.83×10^-02^ | | -0.84 | 0.48 | 8.28×10^-02^ | |
| miR-8069 | 0.65 | 0.32 | 3.95×10^-02^ | | 0.67 | 0.33 | 4.07×10^-02^ | |
| miR-4447 | -0.70 | 0.34 | 3.99×10^-02^ | | -0.66 | 0.34 | 5.18×10^-02^ | |
| miR-140-5p | 1.94 | 0.95 | 4.07×10^-02^ | | 2.09 | 0.96 | 2.95×10^-02^ | |
| miR-4688 | 1.18 | 0.58 | 4.25×10^-02^ | | 1.20 | 0.60 | 4.45×10^-02^ | |
| miR-3124-3p | -0.95 | 0.47 | 4.25×10^-02^ | | -0.85 | 0.50 | 9.07×10^-02^ | |
| miR-4798-5p | -0.80 | 0.40 | 4.37×10^-02^ | | -0.75 | 0.43 | 8.43×10^-02^ | |
| miR-6781-5p | 0.44 | 0.22 | 4.77×10^-02^ | | 0.44 | 0.23 | 5.84×10^-02^ | |
| miR-1225-3p | -2.13 | 1.08 | 4.81×10^-02^ | | -2.07 | 0.98 | 3.51×10^-02^ | |
| miR-6875-5p | 0.53 | 0.27 | 4.95×10^-02^ | | 0.53 | 0.27 | 5.16×10^-02^ | |
| miR-331-3p | 1.58 | 0.83 | 5.75×10^-02^ | | 1.84 | 0.86 | 3.24×10^-02^ | |
| miR-361-5p | 1.74 | 0.91 | 5.72×10^-02^ | | 1.81 | 0.85 | 3.37×10^-02^ | |
| miR-6877-3p | -0.99 | 0.55 | 7.25×10^-02^ | | -1.29 | 0.61 | 3.48×10^-02^ | |
| miR-572 | -0.97 | 0.50 | 5.47×10^-02^ | | -1.13 | 0.54 | 3.73×10^-02^ | |
| miR-590-5p | 1.26 | 0.70 | 7.34×10^-02^ | | 1.48 | 0.72 | 3.94×10^-02^ | |
| miR-128-3p | 1.41 | 0.73 | 5.45×10^-02^ | | 1.62 | 0.79 | 4.01×10^-02^ | |
| miR-378d | 1.43 | 0.77 | 6.42×10^-02^ | | 1.64 | 0.81 | 4.28×10^-02^ | |
| miR-148a-3p | 1.18 | 0.62 | 5.81×10^-02^ | | 1.24 | 0.61 | 4.33×10^-02^ | |
| miR-362-5p | 1.24 | 0.69 | 7.39×10^-02^ | | 1.41 | 0.71 | 4.69×10^-02^ | |
| miR-6731-3p | -1.10 | 0.57 | 5.36×10^-02^ | | -1.18 | 0.60 | 4.85×10^-02^ | |
| miR-6809-3p | -0.96 | 0.49 | 5.08×10^-02^ | | -1.04 | 0.53 | 4.88×10^-02^ | |

Note: Model 1 : adjusted for age, sex, cohort. Model 2: adjusted for model1, smoking status, chronic disease, BMI, RBC, alcohol and education. The table is ranked according to the P values of miRNAs nominally associated (P<0.05) with prevalent haematological tumors in model 1. * in model 2 indicates P < 8.46 × 10−5. Bolded font implies miRNAs significantly associated with incident hematologic tumors. Hematologic tumors refers to lymphatic and hematopoietic tumors. Abbreviations. BMI, body mass index; SE, standard error; RBC, red blood cells; miRNA, microRNA.

**Table S10. Putative target genes of the 13 hematologic tumor-associated miRNAs**

| miRNA id | Gene symbol | miRNA id | Gene symbol | miRNA id | Gene symbol | miRNA id | Gene symbol | miRNA id | Gene symbol | miRNA id | Gene symbol | miRNA id | Gene symbol |
| --- | --- | --- | --- | --- | --- | --- | --- | --- | --- | --- | --- | --- | --- |
| miR-4644 | *HOOK3* | miR-4478 | *MIDEAS* | miR-5196-5p | *CPM* | miR-6870-5p | *STK11* | miR-7111-5p | *NACC1* | miR-4430 | *IDS* | miR-4505 | *C10orf67* |
| miR-4644 | *TMOD2* | miR-4478 | *GOLGA3* | miR-5196-5p | *ANKRD45* | miR-6870-5p | *RAB11FIP4* | miR-7111-5p | *DDX6* | miR-4430 | *ORAI2* | miR-4505 | *CSK* |
| miR-4644 | *MYLK* | miR-4478 | *SOAT1* | miR-5196-5p | *GRB2* | miR-6870-5p | *NACC1* | miR-7111-5p | *UBE2V1* | miR-4430 | *ZNF281* | miR-4505 | *ARHGDIA* |
| miR-4644 | *NR1D1* | miR-4478 | *URM1* | miR-5196-5p | *ARRB2* | miR-6870-5p | *PRELP* | miR-7111-5p | *PSME3* | miR-4430 | *PLXDC2* | miR-4505 | *GTF3C1* |
| miR-4644 | *TLNRD1* | miR-4478 | *CENPN* | miR-5196-5p | *MYH14* | miR-6870-5p | *DDX6* | miR-7111-5p | *SZRD1* | miR-4430 | *SLC35E2B* | miR-4505 | *PCSK6* |
| miR-4644 | *ATP6V1F* | miR-4478 | *MEF2D* | miR-5196-5p | *SP1* | miR-6870-5p | *UBE2V1* | miR-7111-5p | *STMN3* | miR-4430 | *PCSK6* | miR-4505 | *MAP2K7* |
| miR-4644 | *CBX5* | miR-4478 | *CLCC1* | miR-5196-5p | *FOXK1* | miR-6870-5p | *PSME3* | miR-7111-5p | *CCSER2* | miR-4430 | *MAP2K7* | miR-4505 | *MAPK8IP3* |
| miR-4644 | *PTCHD1* | miR-4478 | *TXNRD2* | miR-5196-5p | *SZRD1* | miR-6870-5p | *PTPA* | miR-7111-5p | *SALL2* | miR-4430 | *FHL2* | miR-4505 | *SEC22C* |
| miR-4644 | *USP3* | miR-4478 | *DFFB* | miR-5196-5p | *SNX19* | miR-6870-5p | *STMN3* | miR-7111-5p | *PGPEP1* | miR-4430 | *RNF170* | miR-4505 | *R3HDM4* |
| miR-4644 | *SSBP2* | miR-4478 | *OLFML2A* | miR-5196-5p | *SHISA6* | miR-6870-5p | *SUV39H1* | miR-7111-5p | *MYL12A* | miR-4430 | *TSHZ2* | miR-4505 | *MLX* |
| miR-4644 | *KCNC2* | miR-4478 | *UTP15* | miR-5196-5p | *TNPO2* | miR-6870-5p | *CCSER2* | miR-7111-5p | *CBX6* | miR-4430 | *SEC14L4* | miR-4505 | *LACTB* |
| miR-4644 | *KLF7* | miR-4478 | *ZNF124* | miR-5196-5p | *CASTOR2* | miR-6870-5p | *MAP7D1* | miR-7111-5p | *RAB11FIP4* | miR-4430 | *AGO3* | miR-4505 | *SEPTIN14* |
| miR-4644 | *NFIX* | miR-4478 | *ZNF417* | miR-5196-5p | *URM1* | miR-6870-5p | *CBX6* | miR-7111-5p | *RAB15* | miR-4430 | *CYP20A1* | miR-4505 | *RAP1GAP2* |
| miR-4644 | *PAK6* | miR-4478 | *KIF3A* | miR-5196-5p | *TPM3* | miR-6870-5p | *RAB15* | miR-7111-5p | *ZBTB7A* | miR-4430 | *MPRIP* | miR-4505 | *PAK4* |
| miR-4644 | *MDM4* | miR-4478 | *PGPEP1* | miR-5196-5p | *SMC1A* | miR-6870-5p | *PGPEP1* | miR-7111-5p | *NFAT5* | miR-4430 | *DBT* | miR-4505 | *CDC37* |
| miR-4644 | *NR6A1* | miR-4478 | *MEMO1* | miR-5196-5p | *CAPZB* | miR-6870-5p | *PKM* | miR-7111-5p | *PRRT2* | miR-4430 | *ST3GAL1* | miR-4505 | *TMEM248* |
| miR-4644 | *FAM168A* | miR-4478 | *TMEM170A* | miR-5196-5p | *ITM2C* | miR-6870-5p | *ZBTB7A* | miR-7111-5p | *KCNH2* | miR-4430 | *UNC13A* | miR-4505 | *TMEM63C* |
| miR-4644 | *ZNF385A* | miR-4478 | *TMEM59* | miR-5196-5p | *PDE6D* | miR-6870-5p | *RUNX3* | miR-7111-5p | *ANKRD52* | miR-4430 | *PLEKHM3* | miR-4505 | *PNMA8B* |
| miR-4644 | *TRIM67* | miR-4478 | *EVI5* | miR-5196-5p | *RPRD2* | miR-6870-5p | *HNRNPAB* | miR-7111-5p | *DIPK2B* | miR-4430 | *HEYL* | miR-4505 | *ITGA3* |
| miR-4644 | *TCF12* | miR-4478 | *SNX27* | miR-5196-5p | *ARHGDIA* | miR-6870-5p | *TMEM54* | miR-7111-5p | *PTPA* | miR-4430 | *SIK2* | miR-4505 | *PRKCA* |
| miR-4644 | *EPHB2* | miR-4478 | *HOOK3* | miR-5196-5p | *CYSLTR2* | miR-6870-5p | *LARP1* | miR-7111-5p | *PHC2* | miR-4430 | *METTL2B* | miR-4505 | *NECTIN1* |
| miR-4644 | *AGO1* | miR-4478 | *ZNF587* | miR-5196-5p | *AP2M1* | miR-6870-5p | *ANKRD13B* | miR-7111-5p | *PEDS1-UBE2V1* | miR-4430 | *MAVS* | miR-4505 | *LLGL1* |
| miR-4644 | *PPM1L* | miR-4478 | *ZNF566* | miR-5196-5p | *WDR82* | miR-6870-5p | *KCNH2* | miR-7111-5p | *NFASC* | miR-4430 | *ZFP14* | miR-4505 | *NEURL1* |
| miR-4644 | *LSAMP* | miR-4478 | *ZNF101* | miR-5196-5p | *HNRNPC* | miR-6870-5p | *ANKRD52* | miR-7111-5p | *MTHFR* | miR-4430 | *GTF2F1* | miR-4505 | *RGS6* |
| miR-4644 | *SUPT6H* | miR-4478 | *RFT1* | miR-5196-5p | *MARVELD1* | miR-6870-5p | *DIPK2B* | miR-7111-5p | *UBTF* | miR-4430 | *LYN* | miR-4505 | *NDEL1* |
| miR-4644 | *URM1* | miR-4478 | *MOB3A* | miR-5196-5p | *ABHD14B* | miR-6870-5p | *PEDS1-UBE2V1* | miR-7111-5p | *LARP1* | miR-4430 | *PRKCA* | miR-4505 | *KDM6B* |
| miR-4644 | *ST6GALNAC5* | miR-4478 | *MAPK1IP1L* | miR-5196-5p | *LUZP1* | miR-6870-5p | *MTHFR* | miR-7111-5p | *DIRAS2* | miR-4430 | *PTPN2* | miR-4505 | *CELF1* |
| miR-4644 | *PCDHA1* | miR-4478 | *TNFRSF10B* | miR-5196-5p | *ERI2* | miR-6870-5p | *UBTF* | miR-7111-5p | *SLC48A1* | miR-4430 | *SNX3* | miR-4505 | *ECE1* |
| miR-4644 | *PCDHA4* | miR-4478 | *TPM3* | miR-5196-5p | *HSPB6* | miR-6870-5p | *KMT2D* | miR-7111-5p | *P3H2* | miR-4430 | *ABL2* | miR-4505 | *ACVRL1* |
| miR-4644 | *PCDHA6* | miR-4478 | *ZNF641* | miR-5196-5p | *PRRT2* | miR-6870-5p | *SLC9A3R2* | miR-7111-5p | *HOXC8* | miR-4430 | *BTN3A2* | miR-4505 | *PTGIS* |
| miR-4644 | *PCDHA9* | miR-4478 | *TRIM65* | miR-5196-5p | *ANKRD52* | miR-6870-5p | *DIRAS2* | miR-7111-5p | *TAOK1* | miR-4430 | *GALNT6* | miR-4505 | *IBA57* |
| miR-4644 | *PCDHA10* | miR-4478 | *ZNF619* | miR-5196-5p | *MPRIP* | miR-6870-5p | *RPP25* | miR-7111-5p | *ITGA3* | miR-4430 | *MAFF* | miR-4505 | *ILK* |
| miR-4644 | *LCOR* | miR-4478 | *TMTC1* | miR-5196-5p | *ZMYND11* | miR-6870-5p | *MNT* | miR-7111-5p | *CCL22* | miR-4430 | *XIAP* | miR-4505 | *TMEM184A* |
| miR-4644 | *MYO18A* | miR-4478 | *WFDC8* | miR-5196-5p | *ABHD2* | miR-6870-5p | *TAOK1* | miR-7111-5p | *TFAP2B* | miR-4430 | *SLC26A2* |  |  |
| miR-4644 | *MAPK14* | miR-4478 | *SLC36A2* | miR-5196-5p | *C5orf24* | miR-6870-5p | *HOXB5* | miR-7111-5p | *HNRNPAB* | miR-4430 | *SLC4A1* |  |  |
| miR-4644 | *UHMK1* | miR-4478 | *APAF1* | miR-5196-5p | *CAPN1* | miR-6870-5p | *CCL22* | miR-7111-5p | *ABL1* | miR-4430 | *TIMM50* |  |  |
| miR-4644 | *SGMS1* | miR-4478 | *C1orf174* | miR-5196-5p | *SH3PXD2A* | miR-6870-5p | *SRF* | miR-7111-5p | *SCAMP2* | miR-4430 | *RAB11FIP1* |  |  |
| miR-4644 | *FAM76A* | miR-4478 | *BLOC1S3* | miR-5196-5p | *ZNF516* | miR-6870-5p | *TFAP2B* | miR-7111-5p | *PNPLA6* | miR-4430 | *IBA57* |  |  |
| miR-4644 | *CREBRF* | miR-4478 | *ZNF701* | miR-5196-5p | *ADGRL1* | miR-6870-5p | *CALR* | miR-7111-5p | *BTG2* | miR-4430 | *GK5* |  |  |
| miR-4644 | *NMNAT2* | miR-4478 | *CBFA2T2* | miR-5196-5p | *SBNO1* | miR-6870-5p | *ABL1* | miR-7111-5p | *GDI1* | miR-4430 | *CUBN* |  |  |
| miR-4644 | *DIPK2B* | miR-4478 | *TBC1D24* | miR-5196-5p | *FOXJ2* | miR-6870-5p | *LPCAT3* | miR-7111-5p | *MAT1A* | miR-4430 | *ZNF154* |  |  |
| miR-4644 | *SF1* | miR-4478 | *ENAH* | miR-5196-5p | *CRY2* | miR-6870-5p | *PNPLA6* | miR-7111-5p | *MEX3A* | miR-4430 | *TMEM184A* |  |  |
| miR-4644 | *TANC2* | miR-4478 | *SUMF2* | miR-5196-5p | *PPP1R11* | miR-6870-5p | *BTG2* | miR-7111-5p | *WEE1* | miR-4430 | *ELK1* |  |  |
| miR-4644 | *SPIN3* | miR-4478 | *ANGPT4* | miR-5196-5p | *CSNK1G1* | miR-6870-5p | *GDI1* |  |  |  |  |  |  |
| miR-4644 | *HIC2* | miR-4478 | *CDKAL1* | miR-5196-5p | *MARCKSL1* | miR-6870-5p | *ACTB* |  |  |  |  |  |  |
| miR-4644 | *RSBN1* | miR-4478 | *RNF125* | miR-5196-5p | *UBE2Z* | miR-6870-5p | *P3H2* |  |  |  |  |  |  |
| miR-4644 | *CAMK1G* | miR-4478 | *LIN7C* | miR-5196-5p | *FSCN1* | miR-6870-5p | *CASTOR2* |  |  |  |  |  |  |
| miR-4644 | *CA10* | miR-4478 | *PDP2* | miR-5196-5p | *EIF4EBP2* |  |  |  |  |  |  |  |  |
| miR-4644 | *AAK1* | miR-4478 | *CD209* | miR-5196-5p | *RGS6* |  |  |  |  |  |  |  |  |
| miR-4644 | *ADGRL1* | miR-4478 | *PHACTR4* | miR-5196-5p | *NKX2-5* |  |  |  |  |  |  |  |  |
| miR-4644 | *THSD7A* | miR-4478 | *ZNF329* | miR-5196-5p | *RAB5C* |  |  |  |  |  |  |  |  |
| miR-4644 | *SLC39A14* | miR-4478 | *TSPYL1* | miR-5196-5p | *SHMT2* |  |  |  |  |  |  |  |  |
| miR-4644 | *RIC8B* | miR-4478 | *SLC7A5* | miR-5196-5p | *BTG2* |  |  |  |  |  |  |  |  |
| miR-4644 | *ENAH* | miR-4478 | *PPM1D* | miR-5196-5p | *ERG28* |  |  |  |  |  |  |  |  |
| miR-4644 | *PCDHAC1* | miR-4478 | *MED17* | miR-5196-5p | *GLG1* |  |  |  |  |  |  |  |  |
| miR-4644 | *PCDHAC2* | miR-4478 | *NMUR1* | miR-5196-5p | *CAVIN1* |  |  |  |  |  |  |  |  |
| miR-4644 | *PCDHA11* | miR-4478 | *PPEF2* | miR-5196-5p | *WBP2* |  |  |  |  |  |  |  |  |
| miR-4644 | *PCDHA12* | miR-4478 | *SHOX* | miR-5196-5p | *MSX2* |  |  |  |  |  |  |  |  |
| miR-4644 | *PCDHA13* | miR-4478 | *POLR1G* | miR-5196-5p | *REPIN1* |  |  |  |  |  |  |  |  |
| miR-4644 | *PCDHA2* | miR-4478 | *FBXW2* | miR-5196-5p | *MINK1* |  |  |  |  |  |  |  |  |
| miR-4644 | *PCDHA3* | miR-4478 | *ATM* | miR-5196-5p | *PABPC1L2B* |  |  |  |  |  |  |  |  |
| miR-4644 | *PCDHA5* | miR-4478 | *SLC11A2* | miR-5196-5p | *ZNF436* |  |  |  |  |  |  |  |  |
| miR-4644 | *PCDHA7* | miR-4478 | *NPR1* | miR-5196-5p | *SDK1* |  |  |  |  |  |  |  |  |
| miR-4644 | *PCDHA8* | miR-4478 | *MXRA7* | miR-5196-5p | *NCS1* |  |  |  |  |  |  |  |  |
| miR-4644 | *TEAD1* | miR-4478 | *TRAPPC2* | miR-5196-5p | *AHCYL2* |  |  |  |  |  |  |  |  |
| miR-4644 | *XYLT1* | miR-4478 | *GLUL* |  |  |  |  |  |  |  |  |  |  |
| miR-4644 | *ABCG4* | miR-4478 | *ARSA* |  |  |  |  |  |  |  |  |  |  |
| miR-4644 | *IKZF4* | miR-4478 | *FBXO45* |  |  |  |  |  |  |  |  |  |  |
| miR-4644 | *CDH4* | miR-4478 | *TVP23C* |  |  |  |  |  |  |  |  |  |  |
| miR-4644 | *CTSE* | miR-4478 | *CASTOR2* |  |  |  |  |  |  |  |  |  |  |
| miR-4644 | *SORL1* | miR-4478 | *SHISA9* |  |  |  |  |  |  |  |  |  |  |
| miR-4644 | *PRKAR2A* |  |  |  |  |  |  |  |  |  |  |  |  |
| miR-4644 | *ACTN4* |  |  |  |  |  |  |  |  |  |  |  |  |
| miR-4644 | *GPR12* |  |  |  |  |  |  |  |  |  |  |  |  |
| miR-4644 | *MAT2A* |  |  |  |  |  |  |  |  |  |  |  |  |
| miR-4644 | *CORO2B* |  |  |  |  |  |  |  |  |  |  |  |  |
| miR-4644 | *PITPNA* |  |  |  |  |  |  |  |  |  |  |  |  |
| miR-4644 | *DPF2* |  |  |  |  |  |  |  |  |  |  |  |  |
| miR-4644 | *SLC16A2* |  |  |  |  |  |  |  |  |  |  |  |  |
| miR-4644 | *RERE* |  |  |  |  |  |  |  |  |  |  |  |  |
| miR-4644 | *SEC22A* |  |  |  |  |  |  |  |  |  |  |  |  |
| miR-4644 | *CNTD1* |  |  |  |  |  |  |  |  |  |  |  |  |
| miR-4644 | *SLC8A1* |  |  |  |  |  |  |  |  |  |  |  |  |
| miR-4644 | *CCND2* |  |  |  |  |  |  |  |  |  |  |  |  |
| miR-4644 | *IGF2R* |  |  |  |  |  |  |  |  |  |  |  |  |
| miR-4644 | *ZNF704* |  |  |  |  |  |  |  |  |  |  |  |  |
| miR-4644 | *MYBL1* |  |  |  |  |  |  |  |  |  |  |  |  |
| miR-4644 | *SPATA2* |  |  |  |  |  |  |  |  |  |  |  |  |
| miR-4644 | *ZBTB20* |  |  |  |  |  |  |  |  |  |  |  |  |
| miR-4644 | *SMG7* |  |  |  |  |  |  |  |  |  |  |  |  |
| miR-4644 | *SLC24A2* |  |  |  |  |  |  |  |  |  |  |  |  |
| miR-4644 | *ATP1A3* |  |  |  |  |  |  |  |  |  |  |  |  |
| miR-4644 | *KDM2A* |  |  |  |  |  |  |  |  |  |  |  |  |
| miR-4644 | *CADM1* |  |  |  |  |  |  |  |  |  |  |  |  |
| miR-4644 | *FAM53C* |  |  |  |  |  |  |  |  |  |  |  |  |
| miR-4644 | *CHST11* |  |  |  |  |  |  |  |  |  |  |  |  |
| miRNA id | Gene symbol | miRNA id | Gene symbol | miRNA id | Gene symbol | miRNA id | Gene symbol | miRNA id | Gene symbol | miRNA id | Gene symbol | miRNA id | Gene symbol |
| miR-4534 | *TRIM56* | miR-6124 | *CALM3* | miR-4644 | *UBE2V1* | miR-6778-5p | *E2F6* | miR-1915-3p | *ADM2* | miR-654-5p | *MEF2D* | miR-4292 | *RIMKLA* |
| miR-4534 | *KDM2A* | miR-6124 | *CORO1C* | miR-4644 | *STX6* | miR-6778-5p | *FAM131B* | miR-1915-3p | *OLFML2A* | miR-654-5p | *ITM2C* | miR-4292 | *PSMF1* |
| miR-4534 | *FOSL1* | miR-6124 | *CYB5B* | miR-4644 | *PDZD4* | miR-6778-5p | *HNRNPU* | miR-1915-3p | *SSTR3* | miR-654-5p | *PEA15* | miR-4292 | *NCDN* |
| miR-4534 | *CDK6* | miR-6124 | *ZNF436* | miR-4644 | *ARID1A* | miR-6778-5p | *DNAJC8* | miR-1915-3p | *RUBCNL* | miR-654-5p | *TMEM239* | miR-4292 | *PACS2* |
| miR-4534 | *SLC9A8* | miR-6124 | *LCOR* | miR-4644 | *ZADH2* | miR-6778-5p | *SUSD6* | miR-1915-3p | *ZNF500* | miR-654-5p | *TSPAN3* | miR-4292 | *STX6* |
| miR-4534 | *MAP2K4* | miR-6124 | *PNISR* | miR-4644 | *SYT2* | miR-6778-5p | *CRY2* | miR-1915-3p | *IP6K1* | miR-654-5p | *SETBP1* | miR-4292 | *TNPO2* |
| miR-4534 | *CEP97* | miR-6124 | *PTP4A2* | miR-4644 | *CELF1* | miR-6778-5p | *KLF7* | miR-1915-3p | *C8orf58* | miR-654-5p | *BBC3* | miR-4292 | *CALU* |
| miR-4534 | *KAT6A* | miR-6124 | *FAM241A* | miR-4644 | *PEDS1-UBE2V1* | miR-6778-5p | *CBX6* | miR-1915-3p | *PTGES2* | miR-654-5p | *CNNM4* |  |  |
| miR-4534 | *NIPA2* | miR-6124 | *APCDD1* | miR-4644 | *SBK1* | miR-6778-5p | *MCFD2* | miR-1915-3p | *TMEM184B* | miR-654-5p | *CD276* |  |  |
| miR-4534 | *KLK10* | miR-6124 | *CREBRF* | miR-4644 | *ABCC5* | miR-6778-5p | *PHC2* | miR-1915-3p | *CBX6* | miR-654-5p | *CALM3* |  |  |
| miR-4534 | *TIGIT* | miR-6124 | *CELF1* | miR-4644 | *CTDNEP1* | miR-6778-5p | *KMT2D* | miR-1915-3p | *FKBP15* | miR-654-5p | *CYP4A11* |  |  |
| miR-4534 | *SMYD1* | miR-6124 | *SF1* | miR-4644 | *BNC2* | miR-6778-5p | *ZMIZ1* | miR-1915-3p | *ZDHHC5* | miR-654-5p | *MEX3A* |  |  |
| miR-4534 | *NFIC* | miR-6124 | *PAQR3* | miR-4644 | *WSB2* | miR-6778-5p | *KLC2* | miR-1915-3p | *DPM2* |  |  |  |  |
| miR-4534 | *TMEM214* | miR-6124 | *MAFK* | miR-4644 | *PPP2R1B* | miR-6778-5p | *FBXL18* | miR-1915-3p | *TIRAP* |  |  |  |  |
| miR-4534 | *SRGAP1* | miR-6124 | *TADA2B* | miR-4644 | *RAB5B* | miR-6778-5p | *PTP4A1* | miR-1915-3p | *BZW1* |  |  |  |  |
| miR-4534 | *VPS53* | miR-6124 | *ASAP1* | miR-4644 | *ARHGEF6* | miR-6778-5p | *KHSRP* |  |  |  |  |  |  |
| miR-4534 | *VASH1* | miR-6124 | *DDA1* | miR-4644 | *DLX6* | miR-6778-5p | *HDGF* |  |  |  |  |  |  |
| miR-4534 | *RNF125* | miR-6124 | *TNRC6A* | miR-4644 | *PSME3* | miR-6778-5p | *EFNA3* |  |  |  |  |  |  |
| miR-4534 | *C1orf50* | miR-6124 | *ADAM22* | miR-4644 | *BASP1* | miR-6778-5p | *PLK1* |  |  |  |  |  |  |
| miR-4534 | *PIP4K2C* | miR-6124 | *HAPLN1* | miR-4644 | *BTG2* | miR-6778-5p | *CRTAP* |  |  |  |  |  |  |
| miR-4534 | *PLAGL2* | miR-6124 | *E2F3* | miR-4644 | *USB1* |  |  |  |  |  |  |  |  |
| miR-4534 | *GPRC5A* | miR-6124 | *USP2* | miR-4644 | *AR* |  |  |  |  |  |  |  |  |
| miR-4534 | *SCN2B* | miR-6124 | *NCL* | miR-4644 | *SLC6A17* |  |  |  |  |  |  |  |  |
| miR-4534 | *MLF2* | miR-6124 | *CCDC6* | miR-4644 | *HDX* |  |  |  |  |  |  |  |  |
| miR-4534 | *NAB2* | miR-6124 | *SOD2* |  |  |  |  |  |  |  |  |  |  |
| miR-4534 | *GALNT6* | miR-6124 | *CTNND1* |  |  |  |  |  |  |  |  |  |  |
| miR-4534 | *ALDH9A1* |  |  |  |  |  |  |  |  |  |  |  |  |
| miR-4534 | *FSD2* |  |  |  |  |  |  |  |  |  |  |  |  |
| miR-4534 | *OLA1* |  |  |  |  |  |  |  |  |  |  |  |  |
| miR-4534 | *GLUL* |  |  |  |  |  |  |  |  |  |  |  |  |
| miR-4534 | *CTNND1* |  |  |  |  |  |  |  |  |  |  |  |  |
| miR-4534 | *PPP2R1B* |  |  |  |  |  |  |  |  |  |  |  |  |

**Table S11. Putative target genes of the 13 hematologic tumor-related miRNAs previously linked with hematologic tumors through other (epi)genetic studies**

| GWAS (Trait id: MONDO_0002334) | EWAS (PMID:29933410;26237075; 23722552) |
| --- | --- |
| *CDKAL1* | *CCSER2* |
| *ATM* | *SETBP1* |
| *DDX6* | *SEC14L4* |
| *ERI2* | *TEAD1* |
| *LCOR* | *TFAP2B* |
| *ZBTB7A* | *ARHGEF6* |
| *ZMIZ1* | *BASP1* |
| *TNPO2* | *TNFRSF10B* |
| *NAB2* | *PCDHA1* |
| *TEAD1* | *PCDHA2* |
| *SLC8A1* | *PCDHA3* |
| *ADGRL1* | *PCDHA4* |
| *PTPN2* | *PCDHA5* |
| *FSD2* | *PCDHA6* |
| *PCSK6* | *PCDHA7* |
| *ST6GALNAC5* | *PCDHA8* |
| *SHISA6* | *PCDHA9* |
| *SYT2* | *PCDHA10* |
| *XYLT1* | *PCDHA11* |
| *TNRC6A* | *PCDHA12* |
| *SBK1* | *PCDHA13* |
| *RAB11FIP4* | *ST6GALNAC5* |
| *CCDC6* | *CSNK1G1* |
| *PSMF1* | *SNX27* |
| *MYLK* | *RAB5C* |
| *SLC36A2* | *CSK* |
| *THSD7A* | *HNRNPU* |
| *TMEM248* | *BTG2* |
| *AHCYL2* | *EPHB2* |
| *CAMK1G* | *SLC7A5* |
| *PLXDC2* | *NMNAT2* |
|  | *HOXB5* |
|  | *RAP1GAP2* |
|  | *SALL2* |
|  | *FHL2* |
|  | *SUSD6* |
|  | *TMOD2* |
|  | *TMEM63C* |
|  | *SLC6A17* |
|  | *RERE* |
|  | *KDM6B* |
|  | *SRGAP1* |
|  | *MAPK8IP3* |
|  | *TRIM67* |
|  | *SHISA9* |
|  | *ACTB* |
|  | *KLF7* |
|  | *HIC2* |
|  | *FBXL18* |
|  | *PIP4K2C* |
|  | *PCDHAC1* |
|  | *CADM1* |
|  | *SIK2* |
|  | *MSX2* |
|  | *TCF12* |
|  | *AR* |
|  | *VASH1* |
|  | *ASAP1* |
|  | *NFIC* |
|  | *E2F6* |
|  | *SHISA6* |
|  | *CHST11* |
|  | *SLC35E2B* |
|  | *SF1* |
|  | *DIRAS2* |
|  | *MYH14* |
|  | *GPR12* |
|  | *MAFK* |
|  | *SCAMP2* |
|  | *ST3GAL1* |
|  | *C10orf67* |
|  | *WDR82* |
|  | *ZMIZ1* |
|  | *ABL2* |
|  | *ZBTB20* |
|  | *PLXDC2* |
|  | *FAM168A* |
|  | *CDC37* |
|  | *SDK1* |
|  | *DLX6* |
|  | *LSAMP* |
|  | *ANKRD52* |
|  | *ZNF329* |
|  | *TRAPPC2* |
|  | *WSB2* |
|  | *UBTF* |
|  | *MYLK* |
|  | *CYB5B* |
|  | *SLC4A1* |
|  | *ECE1* |
|  | *KLK10* |
|  | *TMTC1* |
|  | *NFASC* |
|  | *ZNF385A* |
|  | *HEYL* |
|  | *ADAM22* |
|  | *ARID1A* |
|  | *PSME3* |
|  | *DDX6* |
|  | *BNC2* |
|  | *PCDHAC2* |
|  | *WEE1* |
|  | *NPR1* |
|  | *FOXK1* |
|  | *MARCKSL1* |
|  | *NFIX* |
|  | *CDH4* |
|  | *RUNX3* |
|  | *APCDD1* |
|  | *GLG1* |
|  | *CA10* |
|  | *ZNF516* |
|  | *SLC9A3R2* |
|  | *NACC1* |
|  | *PPM1L* |
|  | *PTPN2* |
|  | *RAB15* |
|  | *PDZD4* |
|  | *CBX5* |
|  | *STK11* |
|  | *MPRIP* |
|  | *CORO2B* |
|  | *SLC16A2* |
|  | *ZNF154* |
|  | *VPS53* |
|  | *TXNRD2* |
|  | *TADA2B* |
|  | *PRKCA* |
|  | *IDS* |
|  | *ATP1A3* |
|  | *BBC3* |
|  | *KLC2* |
|  | *KCNC2* |
|  | *GOLGA3* |
|  | *PLAGL2* |
|  | *NFAT5* |
|  | *MXRA7* |
|  | *CDK6* |
|  | *UNC13A* |
|  | *SCN2B* |
|  | *ZNF701* |
|  | *GDI1* |
|  | *RGS6* |
|  | *NKX2-5* |
|  | *P3H2* |
|  | *XIAP* |
|  | *ZNF500* |
|  | *PTGIS* |
|  | *LUZP1* |
|  | *MYO18A* |
|  | *SH3PXD2A* |
|  | *NEURL1* |
|  | *KCNH2* |
|  | *MNT* |
|  | *CAPZB* |
|  | *TVP23C* |
|  | *SHMT2* |
|  | *UBE2V1* |
|  | *FSCN1* |
|  | *PEA15* |
|  | *RIMKLA* |
|  | *SPIN3* |
|  | *SLC48A1* |
|  | *SMG7* |
|  | *MDM4* |
|  | *CLCC1* |
|  | *FOSL1* |
|  | *CELF1* |
|  | *R3HDM4* |
|  | *KAT6A* |
|  | *SLC24A2* |
|  | *PPEF2* |
|  | *CCND2* |
|  | *ANGPT4* |
|  | *ACVRL1* |
|  | *ANKRD45* |
|  | *MAPK14* |
|  | *ZBTB7A* |
|  | *LACTB* |
|  | *CCDC6* |
|  | *ZADH2* |
|  | *MOB3A* |
|  | *MAVS* |
|  | *PTCHD1* |
|  | *PCSK6* |
|  | *EFNA3* |
|  | *SUV39H1* |
|  | *SMYD1* |
|  | *PACS2* |
|  | *MEX3A* |
|  | *C8orf58* |
|  | *C1orf174* |
|  | *KDM2A* |
|  | *ANKRD13B* |
|  | *ORAI2* |
|  | *LYN* |
|  | *SMC1A* |
|  | *REPIN1* |
|  | *PTP4A1* |
|  | *ZNF704* |
|  | *FOXJ2* |
|  | *ITGA3* |
|  | *CPM* |
|  | *RAB11FIP4* |
|  | *CAMK1G* |
|  | *CUBN* |
|  | *CDKAL1* |
|  | *GALNT6* |
|  | *PRELP* |
|  | *NECTIN1* |
|  | *USP2* |
|  | *SGMS1* |
|  | *TAOK1* |
|  | *NDEL1* |
|  | *GRB2* |

Note: Hematological tumor-related GWAS obtained using the GWAS catalogue database using the trait ID in the Experimental Factor Ontology (EFO): MONDO_0002334.

**Table S12. Supporting evidence for the association between the 13 hematologic tumor-related miRNAs and different cancer types reported in previous studies**

| miRNA | cancer types | Species | Tissue types | Main findings | References |
| --- | --- | --- | --- | --- | --- |
| miR-654-5p | MDS | Human | Bone marrow samples and peripheral blood samples | miR-654-5p was found to be overexpressed in 5q- patients compared to controls. | PMID: 21211043 |
| miR-654-5p | cHL | Human | Lymph node tissue | miR-654-5p expression was down-regulated in cHL tissues | PMID: 26951445 |
| miR-654-3p | NKTCL | Human | cell line SNK6 | DNA demethylation in SNK6 cells treated with gemcitabine resulted in upregulation of miR-654-3p expression. | PMID: 31441360 |
| miR-654-3p | AML | Human | Bone marrow samples and AML cell lines (MOLM-13 and AML-193) | Hsa_circ_0079480 regulates cell growth and apoptosis by regulating the hsa_circ_0079480/miR-654-3p/HDGF axis to drive AML progression. | PMID: 33290265 |
| miR-5196 | ALL | Human | Bone marrow and/or peripheral blood samples | The rs10406069 in miR-5196 is highly correlated with the hyperdiploid subtype, which may contribute to leukemia through the effect of SNPs on the expression level of miR-5196, which in turn affects its target genes (e.g., SMC1A). | PMID: 33245684 |
| miR-4505 | MM | Human | Serum samples | The level of serum exosomal miR-4505 was significantly higher in the MM group than in the control group. | PMID: 31102419 |
| miR-4430 | MM | Human | Bone marrow aspirate and/or Blood samples | Comparison of blood or bone marrow samples from MM patients with healthy individuals revealed a majority overexpression of miRNAs in MM patients, including a 9.34-fold change in miR-4430. | PMID: 28458781 |

Abbreviations: cHL, classical Hodgkin lymphoma; NKTCL, extranodal natural killer/T-cell lymphoma; AML, acute myeloid leukemia; MM, multiple myeloma;

MDS, myelodysplastic syndrome; PFS, progression free survival; miRNA, microRNA.

**Supplementary Figures**

Figure S1 Association of plasma microRNA levels with different incident cancers

Figure S2 Venn diagram of nominally associated miRNAs ( P<0.05) in different incident cancer

Figure S3 KEGG pathways depicting pathways regulated by the 13 identified hematological tumor -associated miRNA


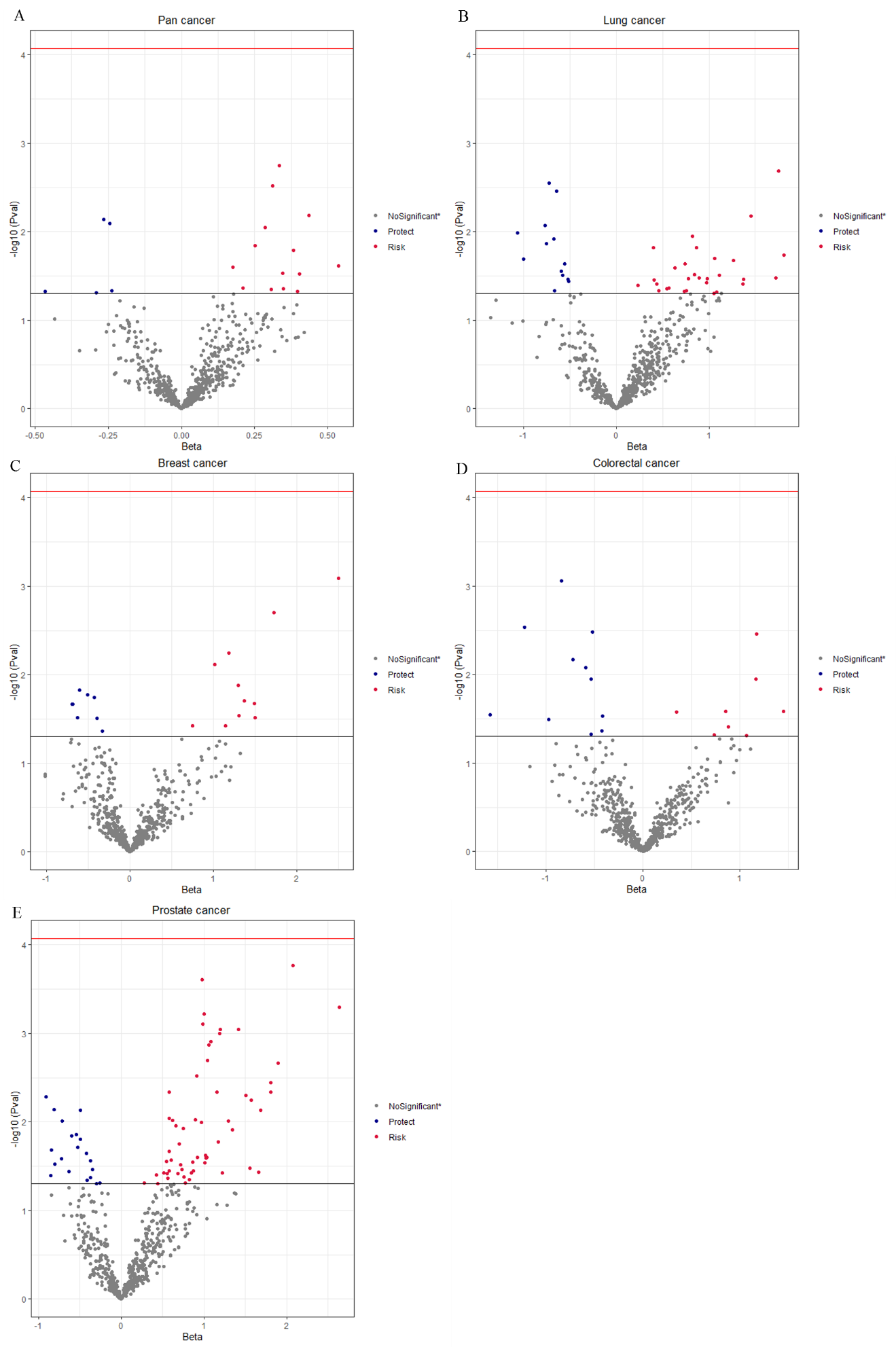


**Figure S1 Association of plasma miRNA levels with different incident cancers**

These Volcano plots depict the results from the Cox proportional hazards regression model. All causes cancer (A), lung cancer (B), breast cancer (C), colorectal cancer (D), and prostate cancer (E). Red dots indicate risk miRNAs that are at least nominally associated. Blue dots are protective miRNAs at least nominally related. Grey dots and * refer to miRNAs with no significant association at *P* < 0.05. Black lines indicate *P* < 0.05, red lines refer to *P* < 8.46 × 10^-5^. Abbreviations: miRNAs, microRNAs.


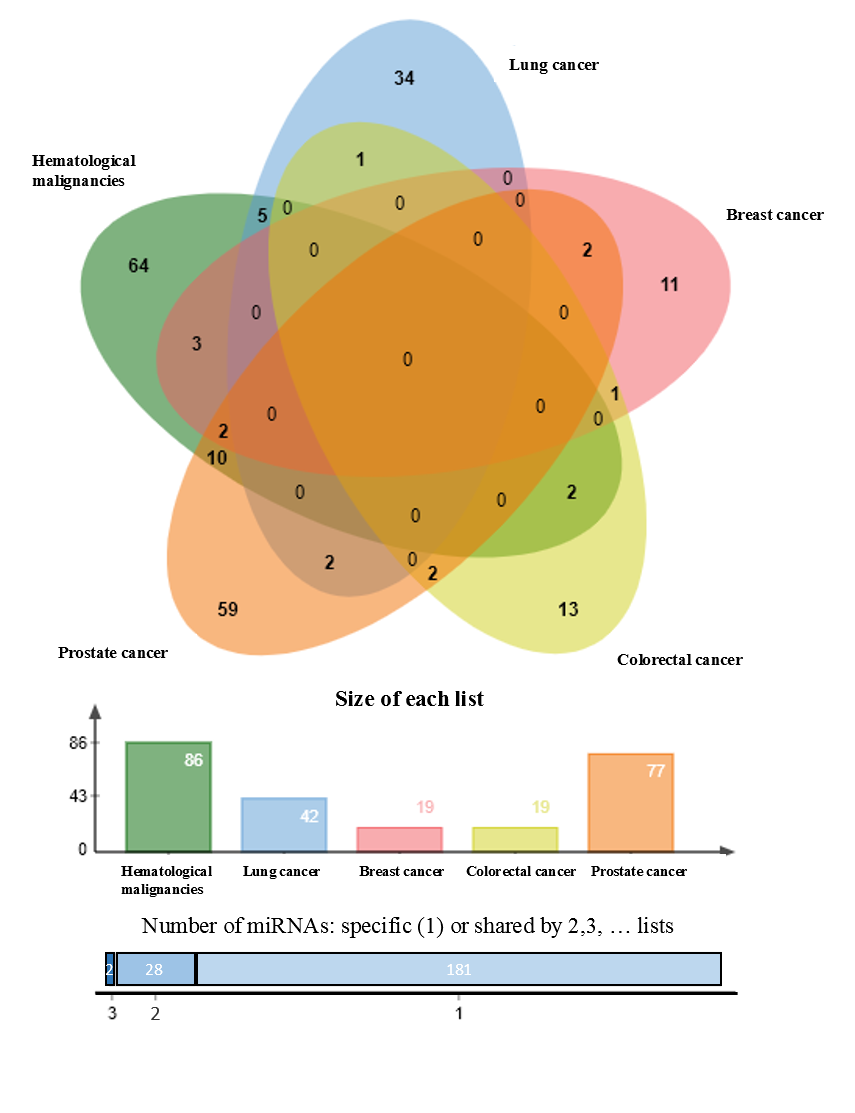


**Figure S2. Venn diagram of nominally associated miRNAs (*P*<0.05) in different incident cancers**

The lower bar suggests the number of nominally associated miRNAs in different incident cancers. The horizontal bar indicates the number of nominally relevant miRNAs that recurred in several cancers. Abbreviations: miRNAs, microRNAs.

**
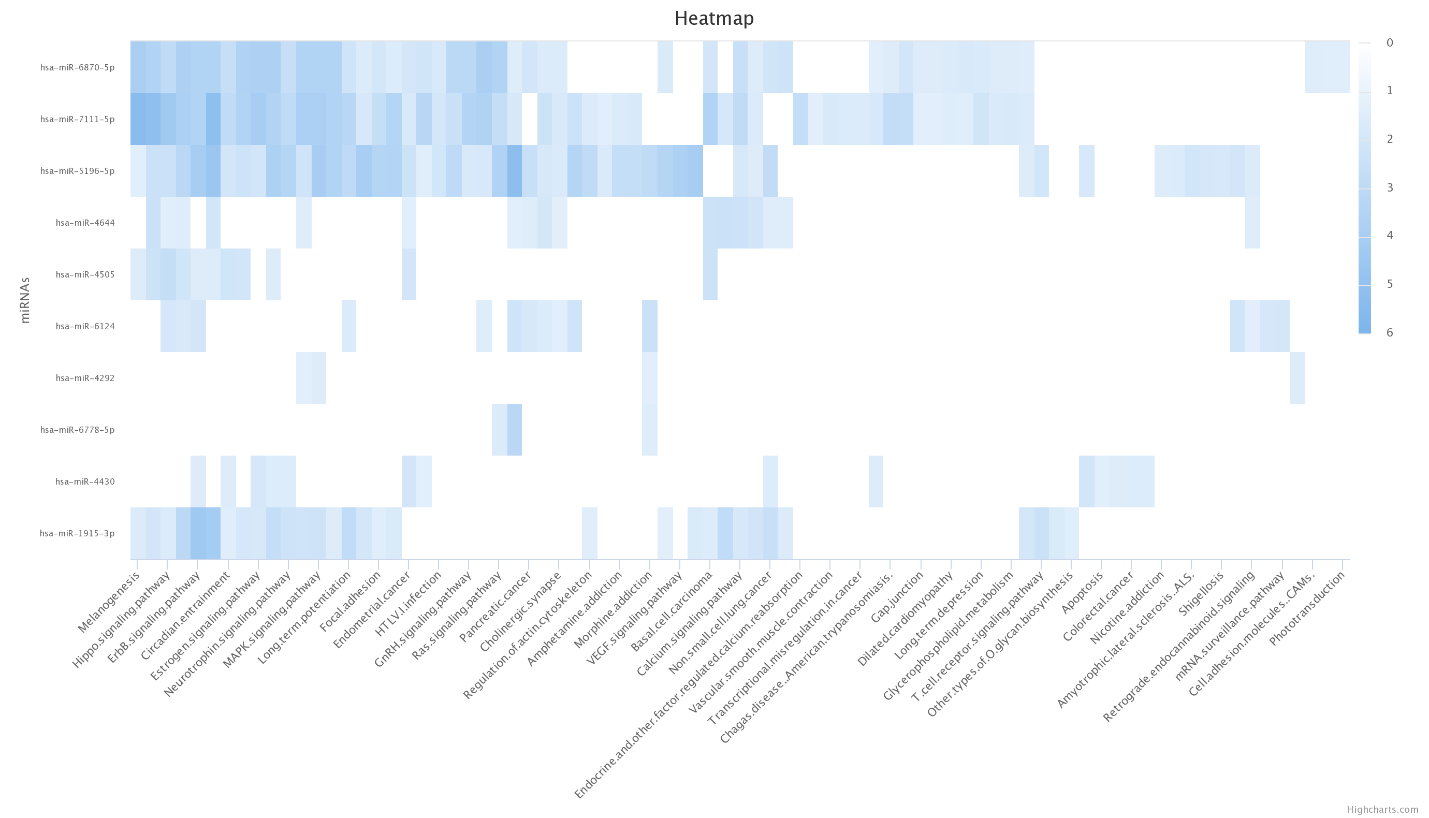
**

**Figure S3. KEGG pathways depicting pathways regulated by the 13 identified hematological tumor-associated miRNAs**

The heatmap depicts enrichment results regulated by 13 identified hematological tumor-associated miRNAs for the categories within KEGG database and predicted miRNA-target interactions. Columns column depicts all KEGG pathways significant for the different miRNAs, while rows illustrate enrichment results for the target genes of hematological tumor-associated miRNAs. Blue color represents the –log10 transformed *P*-value of the enrichment results, the darker the color the more significant the association between given miRNA and target pathway. Abbreviations: miRNAs, microRNAs.

**Supplementary references**

1. Ikram MA, Brusselle G, Ghanbari M, Goedegebure A, Ikram MK, Kavousi M, Kieboom BCT, Klaver CCW, de Knegt RJ, Luik AI *et al*: **Objectives, design and main findings until 2020 from the Rotterdam Study**. *European journal of epidemiology* 2020, **35**(5):483-517.

2. Shah VP, Midha KK, Findlay JW, Hill HM, Hulse JD, McGilveray IJ, McKay G, Miller KJ, Patnaik RN, Powell ML *et al*: **Bioanalytical method validation--a revisit with a decade of progress**. *Pharm Res* 2000, **17**(12):1551-1557.

3. Brämer GR: **International statistical classification of diseases and related health problems. Tenth revision**. *World health statistics quarterly Rapport trimestriel de statistiques sanitaires mondiales* 1988, **41**(1):32-36.

4. Elliott WJ: **Systemic hypertension**. *Curr Probl Cardiol* 2007, **32**(4):201-259.

5. Vijan S: **In the clinic. Type 2 diabetes**. *Ann Intern Med* 2015, **162**(5):ITC1-16.

6. Albus C, Barkhausen J, Fleck E, Haasenritter J, Lindner O, Silber S: **The Diagnosis of Chronic Coronary Heart Disease**. *Dtsch Arztebl Int* 2017, **114**(42):712-719.

7. de Bruijn RF, Bos MJ, Portegies ML, Hofman A, Franco OH, Koudstaal PJ, Ikram MA: **The potential for prevention of dementia across two decades: the prospective, population-based Rotterdam Study**. *BMC Med* 2015, **13**:132.

8. Sticht C, De La Torre C, Parveen A, Gretz N: **miRWalk: An online resource for prediction of microRNA binding sites**. *PLoS One* 2018, **13**(10):e0206239.

9. Agarwal V, Bell GW, Nam JW, Bartel DP: **Predicting effective microRNA target sites in mammalian mRNAs**. *eLife* 2015, **4**.

10. Chen Y, Wang X: **miRDB: an online database for prediction of functional microRNA targets**. *Nucleic acids research* 2020, **48**(D1):D127-d131.

11. Huang HY, Lin YC, Li J, Huang KY, Shrestha S, Hong HC, Tang Y, Chen YG, Jin CN, Yu Y *et al*: **miRTarBase 2020: updates to the experimentally validated microRNA-target interaction database**. *Nucleic acids research* 2020, **48**(D1):D148-d154.

12. Xiong Z, Yang F, Li M, Ma Y, Zhao W, Wang G, Li Z, Zheng X, Zou D, Zong W *et al*: **EWAS Open Platform: integrated data, knowledge and toolkit for epigenome-wide association study**. *Nucleic Acids Res* 2022, **50**(D1):D1004-D1009.

13. Sollis E, Mosaku A, Abid A, Buniello A, Cerezo M, Gil L, Groza T, Güneş O, Hall P, Hayhurst J *et al*: **The NHGRI-EBI GWAS Catalog: knowledgebase and deposition resource**. *Nucleic Acids Res* 2023, **51**(D1):D977-D985.

14. Kehl T, Kern F, Backes C, Fehlmann T, Stöckel D, Meese E, Lenhof HP, Keller A: **miRPathDB 2.0: a novel release of the miRNA Pathway Dictionary Database**. *Nucleic Acids Res* 2020, **48**(D1):D142-D147.

15. Persson H, Kvist A, Rego N, Staaf J, Vallon-Christersson J, Luts L, Loman N, Jonsson G, Naya H, Hoglund M *et al*: **Identification of new microRNAs in paired normal and tumor breast tissue suggests a dual role for the ERBB2/Her2 gene**. *Cancer Res* 2011, **71**(1):78-86.

16. Gassner FJ, Zaborsky N, Feldbacher D, Greil R, Geisberger R: **RNA Editing Alters miRNA Function in Chronic Lymphocytic Leukemia**. *Cancers (Basel)* 2020, **12**(5).

17. Pan J, Tong S, Tang J: **Alteration of microRNA profiles by a novel inhibitor of human La protein in HBV-transformed human hepatoma cells**. *J Med Virol* 2018, **90**(2):255-262.

18. Braoudaki M, Lambrou GI, Giannikou K, Milionis V, Stefanaki K, Birks DK, Prodromou N, Kolialexi A, Kattamis A, Spiliopoulou CA *et al*: **Microrna expression signatures predict patient progression and disease outcome in pediatric embryonal central nervous system neoplasms**. *J Hematol Oncol* 2014, **7**:96.

19. Yi W, Liu J, Qu S, Fan H, Lv Z: **An 8 miRNA-Based Risk Score System for Predicting the Prognosis of Patients With Papillary Thyroid Cancer**. *Technol Cancer Res Treat* 2020, **19**:1533033820965594.
